# BAG3 regulates cilia homeostasis of glioblastoma via its WW domain

**DOI:** 10.1002/biof.2060

**Published:** 2024-04-24

**Authors:** Caterina Roth, Lara Paulini, Marina E. Hoffmann, Thorsten Mosler, Ivan Dikic, Andreas Brunschweiger, Hagen Körschgen, Christian Behl, Benedikt Linder, Donat Kögel

**Affiliations:** ^1^ Department of Neurosurgery, Experimental Neurosurgery University Hospital, Goethe University Frankfurt am Main Germany; ^2^ Institute of Biochemistry II, Goethe University Frankfurt am Main Germany; ^3^ Buchmann Institute for Molecular Life Sciences, Goethe University Frankfurt am Main Germany; ^4^ Institute of Pharmacy and Food Chemistry, Faculty of Chemistry and Pharmacy Julius‐Maximilians‐Universität Würzburg Germany; ^5^ Institute of Pathobiochemistry, University Medical Center of the Johannes Gutenberg University Mainz Germany; ^6^ German Cancer Consortium (DKTK), Partner Site Frankfurt Frankfurt am Main Germany; ^7^ German Cancer Research Center DKFZ Heidelberg Germany

**Keywords:** BAG3, cilia homeostasis, glioblastoma, primary cilia, stemness, WW domain, YAP1, YAP1 signaling

## Abstract

The multidomain protein BAG3 exerts pleiotropic oncogenic functions in many tumor entities including glioblastoma (GBM). Here, we compared BAG3 protein–protein interactions in either adherently cultured or stem‐like cultured U251 GBM cells. In line with BAG3's putative role in regulating stem‐like properties, identified interactors in sphere‐cultured cells included different stem cell markers (SOX2, OLIG2, and NES), while interactomes of adherent BAG3‐proficient cells indicated a shift toward involvement of BAG3 in regulation of cilium assembly (ACTR3 and ARL3). Applying a set of BAG3 deletion constructs we could demonstrate that none of the domains except the WW domain are required for suppression of cilia formation by full‐length BAG3 in U251 and U343 cells. In line with the established regulation of the Hippo pathway by this domain, we could show that the WW mutant fails to rescue YAP1 nuclear translocation. BAG3 depletion reduced activation of a YAP1/AURKA signaling pathway and induction of PLK1. Collectively, our findings point to a complex interaction network of BAG3 with several pathways regulating cilia homeostasis, involving processes related to ciliogenesis and cilium degradation.

## Graphical abstract

Building on our previously identified and novel deciliating function of BAG3, we further decipher the molecular mechanisms of BAG3‐regulated cilia homeostasis in glioblastoma (GBM). Based on identification of BAG3 interactomes and the contribution of different functional domains we now demonstrate that BAG3 regulates cilia homeostasis via its WW domain and modulation of YAP1 activity/downstream YAP1‐dependent signaling. We also provide evidence for a complex interaction network of BAG3 with several pathways regulating cilia homeostasis at the level of ciliogenesis and cilium degradation.

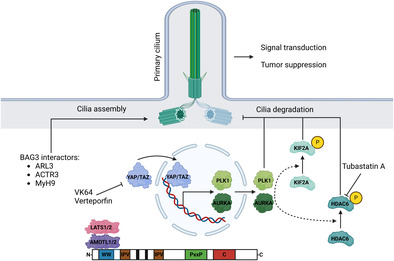

**Abbreviations**
ACTR3: actin related protein 3
ARL3: ADP‐ribosylation factor‐like protein 3
AURKA: aurora kinase A
ANKRD1: ankyrin repeat domain‐containing protein 1
BAG3: BCL2‐associated athanogenic protein 3
CD133: prominin‐1
CSCs: cancer stem cells
CTGF: connective tissue growth factor
CXCR4: CXC motif chemokine receptor 4
CYR61: cysteine‐rich angiogenic inducer 61
EGF: epidermal growth factor
EV: empty vector
FCS: fetal calf serum
FGF: fibroblast growth factor
FL: full‐length
GBM: glioblastoma
GSCs: glioma stem cells
HDAC6: histone deacetylase 6
HSPB8: heat shock protein family B member 8
HSPB1/HSP70: heat shock protein family B (small) member 1
IPV: isoleucine–proline–valine
KO: knock out
LFC: log fold change
MyH9: myosin, heavy chain 9
NEK9: NIMA related kinase 9
NES: nestin
OTC: organotypic tissue culture
OLIG2: oligodendrocyte transcription factor
PLK1: polo‐like‐kinase 1
POU5F1: POU domain, class 5, transcription factor 1
P/S: penicillin–streptomycin
PxxP: proline‐rich domain
SFA: sphere formation assay
SOX2: sex‐determining region Y‐box 2
SOX9: SRY‐box transcription factor 9
STK38: serine/threonine kinase 38
TMZ: temozolomide
TNBC: triple‐negative breast cancer
Tub A: tubastatin A
VIM: vimentin
VP: verteporfin
WT: wild type

## 1 INTRODUCTION

Glioblastoma (GBM) is the most malignant primary tumor of the central nervous system in adults.^1^, ^2^ GBM is categorized as an IDH‐wildtype grade 4 disease,^2^, ^3^, ^4^, ^5^ which has a median survival rate of around 14 months.^1^ Combined with this poor prognosis, the 5‐year survival rate of patients is only 5%. GBM is also characterized by a high resistance to conventional therapies^6^ and strongly infiltrating tumor borders, which are characterized by aggressive growth into the brain parenchyma, making successful treatment including complete surgical resection virtually impossible.^7^, ^8^, ^9^ Surgery, radiotherapy, and chemotherapy with temozolomide (TMZ) are used as standard therapy.^10^ New treatment approaches such as tumor treating fields show small therapeutic successes.^11^ However, prognosis for GBM patients is still dismal and new targeted therapeutic strategies to combat this devastating disease are urgently needed.

The oncoprotein BCL2‐associated athanogenic protein 3 (BAG3) is overexpressed in many types of tumors including GBM^12^, ^13^ and represents an attractive putative target for therapy. BAG3 is induced under stress and, as a multidomain protein, modulates a wide variety of biological processes, like apoptosis and autophagy, cell proliferation, and cytoskeletal dynamics.^13^, ^14^, ^15^ BAG3 contains a BAG domain, a WW domain and a proline‐rich domain (PxxP),^14^ as well as two isoleucine–proline–valine (IPV) motifs.^15^ In addition to its physiological role in protein quality control, BAG3 pathologically regulates a variety of different cancer characteristics, including cell growth and survival, angiogenesis, motility, and metastasis, as well as the enhancement of therapy resistance.^13^, ^14^, ^15^ Previous work by our research group has also shown that overexpression of BAG3 contributes to apoptosis resistance in GBM cells and that depletion of BAG3 in an orthotopic mouse glioma model leads to reduced tumor growth in vivo.^16^ In summary, BAG3 has a potent oncogenic function and acts as a central hub protein promoting multiple cancer hallmarks, which makes it a very interesting therapeutic target.

The primary cilium is an elongated membrane protrusion that is immobile^17^, ^18^ and is formed in a cell cycle‐dependent manner. In addition, primary cilia are present on most mammalian somatic cells.^8^, ^19^ Of note, the presence of cilia is negatively correlated with tumor malignancy, supporting the notion that they act as tumor suppressor organelles.^20^ Ciliogenesis and cilia disassembly are highly complex. They are regulated by cytoskeletal dynamics, autophagy, the cell cycle, as well as by the YAP/TAZ signaling pathway.^8^, ^18^ In return, many signaling pathways are regulated by signals emanating from the cilia. These include the Hedgehog, Wnt, Notch, Hippo, and PDGF pathways, as well as mTOR and several G‐protein‐coupled receptors such as dopamine, serotonin, and melanin receptors.^21^ In addition, primary cilia regulate distinct cellular processes, such as the cell cycle, cytoskeletal dynamics, and cellular proteostasis,^18^ linking cilia to a variety of cancer entities when dysregulated. Defects in primary cilia are referred to as ciliopathies, and many cancer cells have been described as lacking primary cilia.^21^ A variety of cancer features are also associated with a lack of primary cilia, including uncontrolled proliferation, cell metabolism, cell migration and invasion, resistance to cell death, and angiogenesis.^8^ This suggests that impaired ciliogenesis/enhanced turnover of cilia is associated with the aggressiveness of cancer.^22^ Previous work by our group has shown that deletion of BAG3 leads to an increased number of primary cilia in GBM cells and to changes in processes associated with the primary cilium in global proteome/phosphoproteome analysis.^23^ This interesting observation provokes the question inasmuch BAG3's oncogenic functions are mediated by antagonizing the tumor suppressor function of cilia.

The transcription factor YAP1 is a component of the Hippo pathway that serves to inhibit YAP/TAZ. When the Hippo pathway is inactive, YAP/TAZ are dephosphorylated, allowing translocation into the nucleus, where YAP/TAZ can bind to the transcription factors TEAD1‐4 to induce target gene transcription of *Polo‐like‐Kinase 1* (*PLK1*) and *Aurora kinase A* (*AURKA*). The corresponding proteins PLK1 and AURKA exert important roles in cell proliferation, survival, migration, and stemness. If YAP1 is in a phosphorylated state, it is degraded in the cytoplasm.^18^, ^24^ The levels and the activity of YAP/TAZ are elevated in some cancers, including brain tumors,^25^ and increased YAP/TAZ activity is associated with a poor prognosis for patients.^24^, ^25^ Inhibition of ciliogenesis via YAP/TAZ hyperactivity has previously been demonstrated^26^ and may represent a mechanism of YAP/TAZ‐driven cancer hallmarks.

Resistance to therapy is significantly influenced by so‐called cancer stem cells (CSCs), rendering tumors more difficult to treat.^27^, ^28^ These CSCs exhibit a high degree of self‐renewal and are multipotent. CSCs are believed to be largely responsible for the initiation, recurrence, and metastasis of tumors.^29^ CSCs, so‐called glioma stem‐like cells (GSCs), are also found in GBM.^30^ CSCs are characterized by the expression of various proteins associated with stem‐like cells. In GBM, these include the oligodendrocyte transcription factor (OLIG2), Nestin (NES), prominin‐1 (CD133), Nanog, sex‐determining region Y‐Box 2 (SOX2), octamer‐binding transcription factor 4 (POU5F1), CD44,^31^ and SRY‐box transcription factor 9 (SOX9).^32^ Some studies have previously indicated that BAG3 promotes the stem cell character of cancer cells.^33^, ^34^

We have previously identified a novel deciliating function of BAG3 in triple‐negative breast cancer (TNBC) and GBM^23^ that may play an important role in mediating the oncogenic function of BAG3. Cilia homeostasis is regulated both by the frequency of new cilium formation (i.e., ciliogenesis) and by the rate of cilium turnover (cilium degradation) that together regulate the balance in the number of ciliated cells versus unciliated cells in any given culture/organ/tissue.^8^, ^18^ To further decipher the molecular mechanisms of BAG3‐regulated cilia homeostasis in GBM in an unbiased manner, we analyzed BAG3 interactomes and the contribution of different functional domains of BAG3 in the two GBM cell lines U251 and U343. Here, we demonstrate that BAG3 regulates cilia homeostasis via its WW domain and modulation of YAP1 activity/downstream YAP1‐dependent proteins. Further, we provide evidence for a complex interaction network of BAG3 with several pathways regulating cilia homeostasis at the level of ciliogenesis and cilium degradation.

## 2 EXPERIMENTAL PROCEDURES

### 2.1 Cell culture

The experiments were performed with the human GBM cell lines U251‐MG (from now on referred to as U251) and U343,^35^ each with and without BAG3 knock out (KO). The cells were cultured in Dulbecco's Modified Eagle Medium (DMEM) GlutaMAX (Gibco, 61965–026) with 10% fetal calf serum (FCS: Gibco, 10270106) and 100 U/mL penicillin–streptomycin (P/S: Gibco, 15140122). The spheroid cell culture was cultured in Neurobasal‐A medium (Gibco, 10888022) with 2% B‐27 (Gibco, 12587010), 1% GlutaMAX (Gibco, 35050038), 100 U/mL P/S, 20 ng/mL fibroblast growth factor (FGF) (Peprotech, AF‐100‐18B), and epidermal growth factor (EGF) (Peprotech, AF‐100‐15) for at least 72 h. U251 BAG3‐KO cells with the respective BAG3 domain deletion (Del) constructs were cultured in the appropriate medium for adherent or spheroid culture with 1 mg/mL geneticin disulfate (G418; Roth, CP11.3). EGFP positive U251 +/− BAG3‐KO +/− BAG3 domain Del cells were cultured in Neurobasal‐A medium with 5 mg/mL blasticidine S hydrochloride (Sigma, 15,205). All cell lines were passaged twice a week, cultured at 37°C and 5% CO_2_ and tested monthly for mycoplasma contamination. Treatments were performed with 5 μM ABT‐737 (BCL2/BCLxL inhibitor, Santa Cruz Biotechnology, sc‐207242), 5 μM VK64 (TEAD‐YAP inhibitor^36^), 1 μM Verteporfin (VP; TEAD‐YAP inhibitor, MedChemExpress, HY‐B0146), 10 μM Tubastatin A (Cayman, 15785, donated by Ander Matheu, Biodonista Institute, San Sebastián (Spain)), 100 μM TMZ (Sigma‐Aldrich, T2577) for the described time periods.

### 2.2 CRISPR‐Cas9 KOs

Generation of CRISPR/Cas9‐mediated knockout was achieved as described previously^37^ according to published protocols^38^ using a BAG3‐specific guide RNA sequences into pSpCas9(BB)‐2A‐Puro (PX459) as described in Linder et al.^23^

### 2.3 Transfection

U251 BAG3‐KO cells were seeded in 6‐well plates at a cell density of 160,000 cells/well. The next day, transfection with the plasmid DNA for the BAG3 variants or empty vectors (EV) pcDNA3.1 and pcDNA3.1‐FLAG (Addgene, 52535), respectively, with the transfection reagent Invitrogen™ Lipofectamine™ 3000 (Thermo Fisher Scientific, L3000008) was accomplished. The applied BAG3 constructs have already been described previously.^39^, ^40^ Their authenticity was rechecked via Sanger sequencing. All constructs, that is, full‐length (FL) BAG3 (coding for aa 1–575), and different domain deletions of BAG3 like the WW domain Del (not coding for aa 18–53), BAG3 M short domain Del (not coding for aa 97–203), BAG3 M long domain Del (not coding for aa 53–309), BAG3 PxxP domain Del (not coding for aa 321–420) and BAG3 C domain Del (not coding for aa 421–575), comprise an N‐terminal FLAG‐, S‐, SBP‐, and His‐tag. Transfection was performed according to the manufacturer's instructions. Accordingly, 3.75 μL Lipofectamine per 6‐well was diluted in Opti‐MEM and combined with 2.5 μg DNA and 3.75 μL P3000 reagent, also diluted in Opti‐MEM. After 10–15 min incubation at room temperature, the DNA‐lipid complex was added dropwise to the cells and incubated for 8–24 h. Stably transfected cells were selected with 1 mg/mL G418.

### 2.4 Synthesis of lentiviral supernatant and transduction

HEK293T cells were used to produce lentiviral particles and 300,000 cells were seeded in each well of a six‐well plate and incubated overnight at 37°C. Subsequently, the medium of the HEK293T cells was replaced by a transfection medium containing DMEM and 10% FCS without P/S for at least 30 min. Transfection with the plasmid DNA was performed using 2 μg pLV[Exp]‐EGFP:T2A:Bsd‐CMV > ORF_Stuffer (VectorBuilder, Neu‐Isenburg, Germany), 1.5 μg gag/pol plasmid (psPAX2, Addgene #12260) and 0.5 μg VSV‐G envelope plasmid (pMD2. G, Addgene #12259) that were prepared in 57 μL Opti‐MEM (Gibco, 11058–021). The mixture was vortexed and incubated for 10 min. At the same time, 6 μL of Fugene‐HD mix (Promega, Madison, WI, USA) was prepared in 30 μL Opti‐MEM. After that, the Fugene‐HD mix was pipetted onto the DNA mixture and incubated for 30 min at room temperature. After incubation, the mixture was added to HEK293T cells. The plate was incubated at 37°C for 6–8 h, after which the medium was changed to normal DMEM overnight. After 16 and 40 h, the supernatants were collected and pooled with the supernatants from the previous day. The combined supernatant was then filtered with a 0.45 μm filter and the U251 +/− BAG3‐KO cells were transduced. Therefore, 500,000 cells were seeded in a T25 plate and the viral supernatant was added to the corresponding cells in a 1:1 dilution and with 3 μg/mL polybrene (hexadimethrine bromide; Sigma‐Aldrich, 107689). The cells were incubated at 37°C for 24 h. After incubation, the medium was replaced with medium containing 2.5 mg/mL of the selection antibiotic Blasticidine (Thermo Fisher Scientific, Waltham, MA, USA). After 24 h of incubation, the full concentration of the selection antibiotic (5 mg/mL Blasticidine) was added. Cell viability was monitored for 1 week, with at least three washes.

### 2.5 Subcellular fractionation

Subcellular fractionation of cells was performed as previously described.^41^, ^42^ Cell pellets were resuspended in cytosolic extract buffer (CEB; 250 mM sucrose, 70 mM KCl, 137 mM NaCl, 4.3 mM Na_2_HPO_4_, 1.4 mM KH_2_PO_4_). In addition, 400 μg/mL digitonin (Sigma‐Aldrich, St. Louis, MO, USA, D141‐100MG), 100 μM PMSF (Carl Roth GmbH, Karlsruhe, Germany, S367.1), 10 μg/mL leupeptin (AppliChem, Darmstadt, Germany, A2183.0010), and 2 μg/mL aprotinin (Carl Roth GmbH, Karlsruhe, Germany, A162.1) were added to the CEB buffer. The resuspended cells were centrifuged and the supernatant containing the cytoplasmic fraction was removed. The pellet was then incubated with a mitochondrial lysis buffer (MLB; 50 mM Tris–HCl pH 7.4, 150 mM NaCl, 2 mM EDTA, 2 mM EGTA, 0.2% Triton X‐100, 0.3% NP‐40) supplemented with 100 μM PMSF, 10 μg/mL leupeptin and 2 μg/mL aprotinin. And after centrifugation, the mitochondrial fraction was removed. The pellet was then resuspended in RIPA buffer containing HALT phosphatase inhibitors (Thermo Fisher Scientific, Waltham, MA, USA) and complete proteinase inhibitors (Roche, Basel, CHE) and sonicated. After centrifugation, the nuclear fraction was removed. The purity of the fractions was assessed by the presence of GAPDH in the cytosolic fractions and of LAMIN‐A/C in the nuclear extracts using quantitative immunoblots.

### 2.6 Immunoblot‐Analysis

For immunoblot analysis, 120,000 cells per well were seeded in 6‐well plates. The cells were lysed with 2X SDS lysis buffer (137 mM Tris–HCl pH 6.8 (Sigma‐Aldrich, T1503), 4% SDS (Carl Roth, CN30), 20% glycerol (Sigma‐Aldrich, G5516)) including 1:10 phosphatase inhibitor (Roche, 04906837001) and 1:100 protease inhibitor cocktail (Sigma‐Aldrich, P8340) with subsequent sonification. The amount of protein was determined using a Pierce BCA Protein Assay Kit (Thermo Fisher Scientific, 23,225). SDS gels (8%–15% depending on protein size) were loaded with 50 μg protein in 5X SDS loading buffer (250 mM Tris–HCl, pH 6.8, 10% SDS, 30% glycerol, 0.02% bromophenol blue (Sigma‐Aldrich, B8026), 5% 2‐mercaptoethanol (Sigma‐Aldrich, M6250)), after heating the samples to 95°C for 5 min. The proteins were separated (85 V for 30 min, then 125 V for 1–1.5 h) and blotted semidry (15 V, 35 min) onto a nitrocellulose membrane (Cytiva). The membranes were blocked with 5% milk (Carl Roth, T145) or 5% BSA (Carl Roth, 8076) in TBS (150 mM NaCl (Sigma‐Aldrich, 31,434), 50 mM Tris (Sigma, T1503), filled up to 1 L with H_2_O, pH 7.5) with 0.05% Tween‐20 (AppliChem, A4974) (TBS‐T) for 60 min at room temperature, followed by incubation with the primary antibody overnight at 4°C: rabbit‐anti‐BAG3 (Biozol/Abnova, PAB0330, 1:5000), rabbit‐anti‐FLAG (Sigma, F7425, 1:400), rabbit‐anti‐LAMIN‐A/C (Abcam, Cambridge, UK, ab169532, 1:1000), mouse‐anti‐YAP1 (Santa Cruz, sc‐101,199, 1:200), mouse‐anti‐GAPDH (Calbiochem, CB1001, 1:10,000), and mouse‐anti‐vinculin (EMD Milipore, MAB3574, 1:1000). The secondary antibodies (Goat anti‐mouse IRDye 680RD [926–32,220, RRID:AB_10956588], Goat anti‐mouse IRDye 800CW [926–32,210], goat anti‐rabbit IRDye 800CW [926–32,211, RRID:AB_621843], goat anti‐rabbit IRDye 680RD [926–32,221], LI‐COR Bioscience, 1:10,000) were incubated for 1 h at room temperature and then detected with the LI‐Cor Odyssey scanner (LI‐Cor Bioscience).

### 2.7 Immunofluorescence

10,000 U251 and U343 cells were seeded for YAP1 and IFT88 staining was performed with 120,000 U251 cells and 150,000 U343 cells, used per well for 8‐well chamber slides (Thermo Fisher Scientific). After 48 h, the cells were fixed with 4% paraformaldehyde (Santa Cruz Biotechnology, sc‐281,692) for 20 min, followed by three washes with PBS containing 0.01% Tween (AppliChem GmbH, A4974) (PBS‐T). For immunofluorescence staining, cells were permeabilized with 0.5% Triton X‐100 (Sigma‐Aldrich, T8787) in PBS for 5 min and blocked with 4% BSA containing 0.1% Tween in PBS for 1 h at room temperature. Cells were incubated with an antibody against YAP1 (anti‐mouse, Santa Cruz, sc‐101,199, 1:250) or IFT88 (anti‐Rabbit, Proteintech, 13,967‐1‐AP, 1:100) overnight at 4°C, followed by three washes with PBS‐T and subsequent incubation with the secondary antibody Alexa Fluor 488 goat anti‐mouse IgG (H + L; Invitrogen, A11017, 1:500) or antibody Alexa Fluor 488 goat anti‐Rabbit IgG (H + L; Invitrogen, A11070, 1:500) for 1 h at room temperature. For YAP1 counterstaining, incubation with phalloidin 1:40 (Invitrogen, T7471) was performed simultaneously with the secondary antibody. After a further three washes, the slides were mounted with mounting medium Fluoroshield containing DAPI (Sigma‐Aldrich, F6057). The slides were then imaged using a Nikon Eclipse TE2000‐S inverted fluorescence microscope with NIS Elements AR version 4.2 (both Nikon Instruments Europe B.V.) at 60× magnification. For YAP1, five vision fields and for IFT88 10 vision fields per condition were counted. Image processing was performed with ImageJ Fiji V1.52p. For sphere cultures, coating with laminin (Sigma‐Aldrich, L2020) before seeding is necessary to allow GSCs to grow as adherent monolayers maintaining stem‐like properties.^43^, ^44^ 150 μL of a 10 μg/mL solution of laminin in PBS was added per well of an 8‐well chamber slide for 24 h at 4°C.

### 2.8 Sphere formation assay

The sphere formation assay (SFA) was used to determine the changes in sphere area/number and stem‐like properties of GSCs after BAG3 depletion. For this purpose, cells were seeded at a density of 500 cells per well in a 96‐well plate. Each condition was seeded in 10–12 replicates and the cells were incubated for 7 days at 37°C in an incubator. The single wells were photographed with a Tecan Spark plate reader (Tecan). The images of the spheres per well were analyzed with ImageJ Fiji V1.52p. The spheres were counted with a self‐created macro for Fiji. The mean sphere area and the number of spheres were determined for the wild type (WT) and BAG3‐KO for method validation.

### 2.9 Limiting dilution assay

The limiting dilution assay was performed as previously described.^45^, ^46^ Briefly summarized, a flat bottom 96 well plate was used and filled with 200 μL Neurobasal‐A (NBA) medium. A serial dilution was performed starting with 1024, 512, 256, 128, 64, 32, 16, and 8 cells/well for both cell lines U251 and U343 +/− BAG3‐KO. For each cell line, 12 replicates were seeded for each cell dilution. The cells were incubated for 7 days and analyzed with the Nikon Eclipse TE2000‐S inverted microscope with NIS Elements AR version 4.2 (both Nikon Instruments Europe B.V.) at 4× magnification. Stem‐cell frequencies were assessed 7 days after seeding using extreme limiting dilution analysis (ELDA) software using the standard settings (http://bioinf.wehi.edu.au/software/elda ^47^; accessed on February 29, 2024).

### 2.10 Quantitative real‐time polymerase chain reaction (qRT‐PCR)

For the determination of gene expression by qRT‐PCR, triplicates of 300,000 cells per well were seeded in a 6‐well plate for 48 h. After washing the cell pellets with PBS, RNA was isolated using the EXTRACTME total RNA kit according to the manufacturer's instructions (7Bioscience, Hartheim, Germany; https://blirt.eu/wp-content/uploads/2018/08/TOTAL-RNA_protocol_en_29012018_druk_v2-min.pdf). The RNA concentration was measured with the Tecan Spark and 1 μg RNA was used for cDNA synthesis. Vials were filled up with DEPC water to a volume of 11 μL per sample. In addition, 1 μL of 50 μM oligo(dT)20, 1 μL of random hexamer primer (3 μg/μL) and 1 μL of 10 mM dNTP mix were added to each of the RNA mixtures. The mixture was incubated in the Eppendorf cycler (Eppendorf AG, Hamburg, Germany) at 65°C for 5 min and then cooled on ice for 1 min. After addition of a mixture of Superscript III enzyme (200 U; Invitrogen, Carlsbad, USA), DEPC‐H_2_O, 0.1 M DTT and 5× first‐strand buffer, the samples were heated again for 5 min at 25°C. The same mixture was further incubated for 1 h at 50°C and finally incubated for 15 min at 70°C. The resulting cDNA was diluted with 180 μL DEPC water and used directly for qRT‐PCR. The following primers (TaqMan probes were purchased from Thermo Fischer) were used: Hs00427620_m1 (TBP), Hs01053049_s1 (SOX2), Hs04187831_g1 (Nestin), Hs00300164_s1 (OLIG2), Hs00409821_g1 (BMI1), Hs04260367_gH (POU5F1), Hs00165814_m1 (SOX9), Hs00607978_s1 (CXCR4), Hs02387400_g1 (NANOG), Hs00983227_m1 (PLK1), Hs01582072_m1 (AURKA), Hs00173317_m1 (ANKRD1), Hs00155479_m1 (CYR61), Hs00170014_m1 (CTGF). The corresponding primer of the gene of interest, the FastStart master mix and DEPC water were added to the cDNA. The samples were pipetted into a micro‐AMP fast 96‐well reaction plate. The plate was then sealed with an adhesive foil and analyzed using the StepOnePlus qPCR instrument (Applied Biosciences, Foster City, CA, USA). Quantification of gene expression was performed using Graphpad Prism 9 (GraphPad Software, Inc., La Jolla, CA, USA) based on the ΔΔCT method. To this end, samples were corrected to baseline by subtracting the housekeeper gene (TBP) and the expression was shown as log fold change.

### 2.11 Flow cytometric analysis of cell death

The flow cytometric measurements were performed as previously described.^48^ 20,000 cells were seeded in 24‐well plates and treated as described. Cells were transferred with supernatant into FACS tubes and incubated for cell death analysis with 50 μL FACS buffer (10 mM HEPES (AppliChem GmbH, A1069); 140 mM NaCl; 5 mM CaCl_2_ (Merck KGaA, Darmstadt, Germany, 1,023,821,000); pH 7. 4) including 0.8 μL propidium iodide (PI; Ex‐Max: 535 nm, Em‐Max: 617 nm; Sigma‐Aldrich, 236 P4864) and incubated for 10 min at room temperature. The subsequent measurement was performed in the FL3 and FL4 channels with the flow cytometer BD Accuri C6 (BD Biosciences, 488 nm and 640 nm laser and four optical filters (FL1: 533/30 nm, FL2: 585/40 nm, FL3: >670 nm, FL4 675/25 nm)).

### 2.12 Organotypic brain slice culture and ex vivo tumor growth assay

For organotypic tissue culture (OTC), the brains of adult 8–12 week old mice were dissected and the dura mater was removed as previously described.^45^ Mouse brains were then embedded in warm (35°C–37°C) 2% low‐melting agarose (Carl Roth, 6351). A VT1000 vibratome (Leica, Wetzlar, Germany) was used to make 150 μm thick transverse sections. The sections were placed with a brush on Millicell cell culture inserts (Merck KGaA, PICM0RG50) in 6‐well plates and cultured in OTC special medium DMEM/F12 without FCS (Gibco, 31,331,028) with 1× B27, 1× N2 (Gibco, 1,750,248), and 1% P/S (all from Gibco). On the following day, the medium was changed and several of the EGFP U251 +/− BAG3‐KO spheres were placed on the sections and allowed to attach for 1 day. Appropriate spheres were prepared by seeding 1500 cells/wells into U‐shaped 96‐well plates with 200 μL medium and cultured for 72 h. On the following day, baseline images of the tumors (day 0) were taken, and treatment was started (as described in the Figure legends). The respective treatment was renewed three times per week and tumor growth was evaluated according to the duration of the experiment after regular imaging with a Nikon SMZ25 stereomicroscope (Nikon, Minato, Prefecture, Tokyo, Japan) equipped with a P2‐SHR Plan Apo 2× objective and operated with NIS elements software (Nikon software version 4.20). Tissue integrity of mouse brain slices was validated by PI staining (Sigma‐Aldrich, #P4864), with negligible cell death in the tissue slices. Tumor growth was assessed using ImageJ FIJI V1.52p and tumor size was normalized to the baseline size at day 0. The experiments in this study with mouse brains from C57BL6j mice are, according to the German Animal Welfare Act, sacrificing for scientific purposes and do not require prior authorization, but a notification to the authority in charge (Regierungspräsidium Darmstadt). C57BL6j were kept in type II long cages, obtained via Envigo and monitored by the animal welfare officer and central research facility of the Goethe University Hospital.

### 2.13 Interactome sample preparation and data analysis

#### 2.13.1 Cell harvest and lysis

U251 +/‐BAG3‐KO (FLAG‐tagged EV and FL BAG3) cells were seeded in a T75 cell culture flasks at a cell density of 2,000,000 cells per flask. Cells were cultured in DMEM GlutaMAX for adherent culture or in Neurobasal‐A medium for spheroid culture for 72 h. After cultivation cells were pelleted and washed with PBS. Afterwards, cell lysis was performed according to the ChromoTek DYKDDDDK Fab‐Trap™ (FLAG‐Trap) agarose protocol. For lysis the cell pellets were resuspended in 200 μL of ice‐cold lysis buffer (modified RIPA buffer, 10 mM Tris pH 7.5, 150 mM NaCl, 0.5 mM EDTA, 1% Nonident™ P40 Substitute, 1% Triton X‐100, 1% Sodium Deoxycholate, 1% SDS, and filled up with MilliQ) containing protease (1/100) and phosphatase (1/10) inhibitors. After incubation on ice for 30 min the cell lysates were centrifuged at 17,000 x g for 10 min at 4°C and 300 μL of dilution buffer (10 mM Tris pH 7.5, 150 mM NaCl, 0.5 mM EDTA, filled up with MilliQ) containing protease (1/100) and phosphatase (1/10) inhibitors was added to the lysates. Protein concentration was determined with the BCA protein assay kit using 500 μg for the pull‐down (IP) lysate. The rest of the diluted lysate was kept as an input fraction (I) for further analysis.

#### 2.13.2 Pull‐down and sample preparation

For the pull‐down, 25 μL of the FLAG‐Trap bead suspension was equilibrated with 500 μL of ice‐cooled dilution buffer and sedimented by centrifugation at 2500g for 5 min at 4°C. The appropriately diluted protein lysate was added to the equilibrated beads for binding of the FLAG‐tagged protein to the FLAG‐Trap affinity beads at 4°C under end‐to‐end rotation for 1 h. Beads were centrifuged at 2500g for 5 min at 4°C, whereby 50 μL of supernatant was kept for further analysis representing the unbound fraction (N). Subsequently, washing steps were performed with 500 μL of wash buffer (10 mM Tris pH 7.5, 150 mM NaCl, 0.5 mM EDTA, 0.05% Nonidet™ P40 Substitute and filled up with MilliQ), centrifuged at 2500g for 5 min at 4°C, and 50 μL of the supernatant was stored for further analysis representing the flow through fraction (W1‐3). For mass spectrometric analyses, 250 μL of the bead's suspension was centrifuged at 2500g for 5 min at 4°C and the supernatant was discarded and stored on dry ice. The actual interactome experiment was performed in triplicates. The rest of the beads were then resuspended in 80 μL of 2X SDS sample buffer (120 mM Tris pH 6.8, 4% SDS, 20% Glycerol, 0.04% bromophenol blue, 10% β‐Mercaptoethanol and filled up with MilliQ), boiled at 95°C for 5 min, and centrifuged at 4°C for 2 min at 2500g. The supernatant was then analyzed by Western blot analysis to detect the pull‐down efficiency of the FLAG‐BAG3 fusion protein (IP).

#### 2.13.3 LC–MS analyses

Samples were analyzed on a Q Exactive HF coupled to an easy nLC 1200 (ThermoFisher Scientific) using a 35 cm long, 75 μm ID fused‐silica column packed in‐house with 1.9 μm C18 particles (Reprosil pur, Dr. Maisch), and kept at 50°C using an integrated column oven (Sonation). Peptides were eluted by a nonlinear gradient from 4% to 28% acetonitrile over 45 min and directly sprayed into the mass‐spectrometer equipped with a nanoFlex ion source (ThermoFisher Scientific). Full scan MS spectra (350–1650 m/z) were acquired in Profile mode at a resolution of 60,000 at m/z 200, a maximum injection time of 20 ms and an AGC target value of 3 × 10^6^ charges. Up to 10 most intense peptides per full scan were isolated using a 1.4 Th window and fragmented using higher energy collisional dissociation (normalized collision energy of 27). MS/MS spectra were acquired in centroid mode with a resolution of 30,000, a maximum injection time of 110 ms and an AGC target value of 1 × 10^5^. Single‐charged ions, ions with a charge state above 5 and ions with unassigned charge states were not considered for fragmentation and dynamic exclusion was set to 20 s.

#### 2.13.4 Mass spectrometry data processing

MS raw data processing was performed with MaxQuant (v 1.6.17.0) and its in‐build label‐free quantification algorithm MaxLFQ applying default parameters.^49^ Acquired spectra were searched against the human reference proteome (Taxonomy ID 9606) downloaded from UniProt (2023‐02‐14; 20,594 sequences without isoforms) and a collection of common contaminants (244 entries) using the Andromeda search engine integrated in MaxQuant.^50^ Identifications were filtered to obtain false discovery rates below 1% for both peptide spectrum matches (minimum length of seven amino acids) and proteins using a target‐decoy strategy.^51^ The mass spectrometry proteomics data have been deposited to the ProteomeXchange Consortium^52^ via the PRIDE partner repository^53^ with the dataset identifier PXD050279. Further statistical analysis was performed in Perseus (1.6.15.0) and R Bioconductor packages.^49^

#### 2.13.5 Mapping and analysis

Raw data processing and statistical analysis was followed by analysis of interactome data for gene ontology (GO) terms performed using the tool EnrichR.^54^, ^55^ The comparison of sphere and adherent FL BAG3 U251 cells was made with Perseus (1.6.15.0) and the graph with R.

### 2.14 Statistics

GraphPad Prism 9 (GraphPad software) was used for statistical analysis. The data are presented as mean ± SEM. The applied test is stated in the respective figure legend. Changes were considered as significant if *p* ≤ 0.05 and depicted as: *, *p* ≤ 0.01: **, *p* ≤ 0.001: ***, *p* ≤ 0.0001: ****.

## 3 RESULTS

### 3.1 BAG3‐KO reduces stem‐like properties of GSCs

**FIGURE 1:**
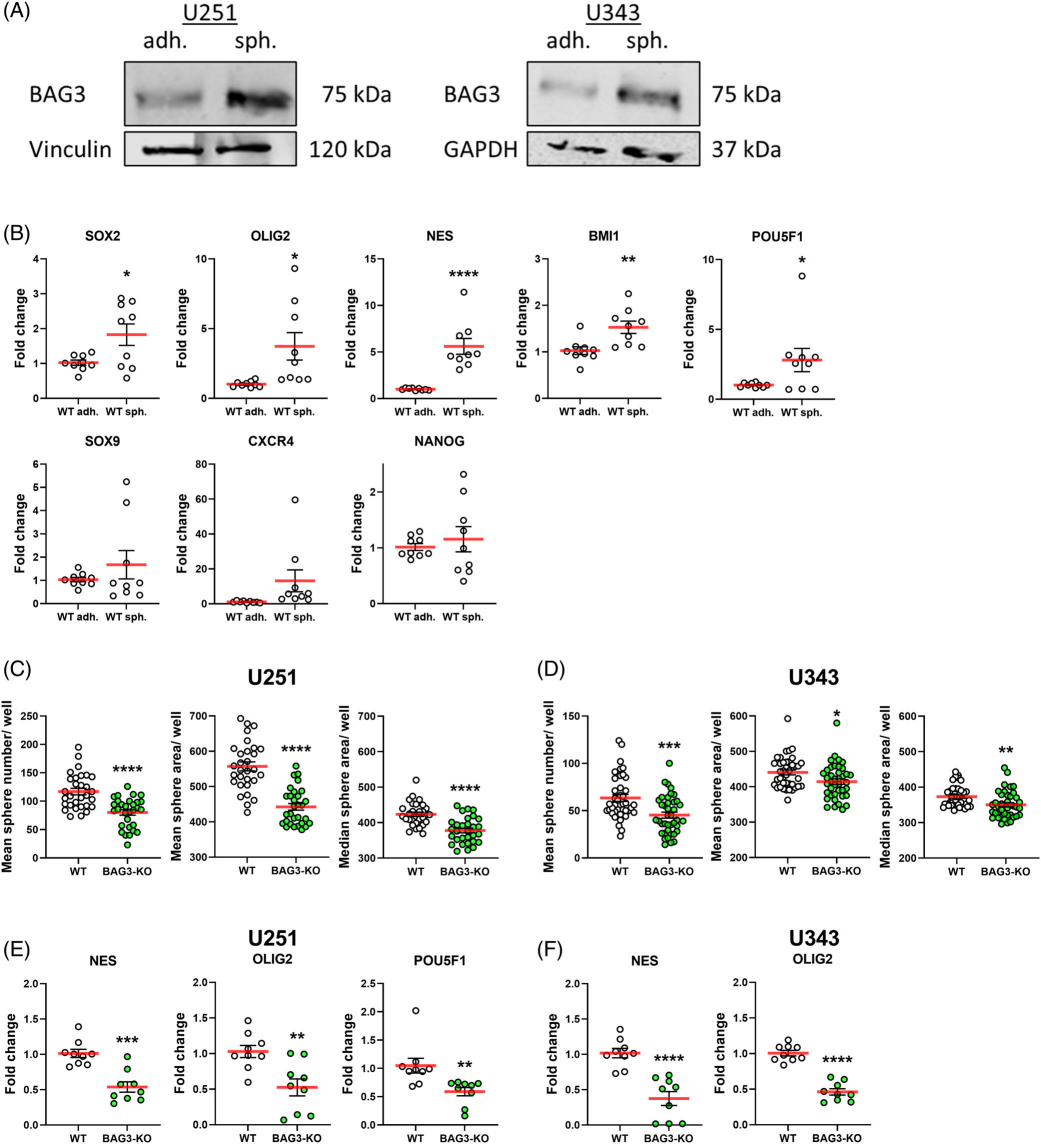
Stemness‐like character of glioblastoma (GBM) cells decreases after BCL2‐associated athanogenic protein 3 (BAG3)‐knock out (KO). Western Blot analysis of the (A) U251 and U343 WT cells cultured in Dulbecco's Modified Eagle Medium (DMEM) (WT adh.) or in Neurobasal‐A medium (NBA) (WT sph.) for 72 h. GAPDH or vinculin served as housekeepers. (B) Taqman‐based quantitative reverse‐transcription polymerase chain reaction of U251 Wild type (WT) cells cultured in DMEM versus NBA medium for 72 h. Diagrams represent data from *n* = 9 replicates with individual gene expression levels of different stemness markers after baseline correction to the reference gene TBP. Unpaired *t*‐test was performed. (C, D) Point plots of the sphere formation assay of (C) U251 and (D) U343 WT and BAG3‐KO GBM cell lines at day 7. Shown from left to right are the mean sphere number/ well, the mean sphere area/ well and the median sphere area/ well of (C) three and (D) four independent experiments. Unpaired t‐test was performed. (E, F) Taqman‐based quantitative reverse‐transcription polymerase chain reaction of (E) U251 and (F) U343 GBM cells after BAG3‐KO of stemness associated marker of three independent experiments, after culturing the cells for 72 h in NBA medium. Single gene expression values are shown as dots (*n* = 9 replicates) after correction to the reference gene TBP. Unpaired *t*‐test was performed. *p < 0.05; **p < 0.01; ***p < 0.001; ****p < 0.0001.

CSCs can be found in many tumors including GBM,^30^ where GSCs exhibit the typical characteristics that lead to therapy resistance and tumor recurrence.^28^ Previous studies suggest that BAG3 is capable to promote a stem‐like cell phenotype in different cancer entities including GBM, correlated to the observation of elevated BAG3 levels in GSCs.^15^, ^33^, ^34^, ^56^ When cultured in spheroid cell medium as tumor spheres, U251 cells exhibit increased levels of endogenous BAG3 (Figure 1A). To correlate these findings with stem‐like properties, U251 BAG3‐KO and WT cells were cultured as spheres for at least 72 h in NBA medium. In line with the results from Tang et al., we confirmed that the U251 WT cell line is a valid model to distinguish a GSC‐like from a more differentiated cell state (Figure 1B).^57^ Stem cell markers such as SOX2, OLIG2, NES, BMI1, and POU5F1 were significantly upregulated when U251 cells were cultured in NBA medium instead of DMEM. Additional markers such as SOX9, CXC motif chemokine receptor 4 (CXCR4), and NANOG were also increased, albeit to a lesser degree (Figure 1B). To further analyze the potential role of BAG3 in transition between these cell states, the U251 and U343 GBM cell lines were cultivated for at least 72 h in sphere NBA medium and a SFA with stable BAG3‐KO cells was used to determine their residual stemness‐like character. Statistical quantification showed a significant reduction of sphere‐forming capacity after BAG3‐KO for the cell lines U251 (Figure 1C) and U343 (Figure 1D), as indicated by a reduced mean sphere number per well. The mean sphere areas in BAG3‐KO control cells were also found to be significantly decreased. To further support the role of BAG3 in regulation of stem‐like properties, we also performed limiting dilution assays. After 7 days of seeding, individual wells were analyzed for the presence of at least one sphere larger than eight cells. We observed that BAG3‐KO significantly reduced the sphere‐forming potential (“stem cell frequency”) of U251 cells, with a change in stem cell frequency from 1:14.7 for the WT to 1:53.3 for the BAG3‐KO cells (Figure S1A,C). A similar finding was observed for the U343 cell line, with a reduction in stem cell frequency from 1:21.2 for the WT to 1:61.3 for the BAG3‐KO cells (Figure S1B,C). To corroborate these results, selected stem cell markers for GBM were analyzed by qPCR. For the U251 cell line, a significant reduction of the stem cell markers NES, OLIG2 and POU5F1 was observed after BAG3 depletion (Figure 1E). Corresponding findings are shown for the U343 cell line (Figure 1F). Overall, our data suggests that BAG3 promotes the stem‐like phenotype of GSCs.

In comparison to normal tumor cells, CSCs are less sensitive to activation of cell death^58^ and previous studies from our group also demonstrated that BAG3 exerts pronounced antiapoptotic effects, as demonstrated by increased resistance to the BH3 mimetic ABT‐737 (Venetoclax) in therapy‐resistant TNBC^59^ and in GBM,^16^ the latter finding now being confirmed in an organotypic brain slice transplantation model of GBM (Figure S2A–C). In line with the limiting effects of BAG3 on tumor growth in vivo,^16^ tumors of BAG3‐depleted U251 cells displayed a pronounced decrease in size increases over time (~3 weeks), while ABT‐737 treatment led to the complete elimination of U251‐BAG3 KO tumors. Furthermore, we could show that treatment with 100 μM TMZ over 96 h leads to robust cell demise in U251 WT and BAG3‐KO cells (Figure S2D), with BAG3 KO cells displaying significantly increased cell death compared with WT cells. As TMZ is a standard therapy for GBM,^10^ this finding further support the suitability of BAG3 as a therapeutic target. Given the pleiotropic functions of BAG3 in various cancer hallmarks,^13^, ^14^, ^15^ we now aimed to further analyze the molecular processes underlying BAG3‐driven malignancy in GBM, including domain‐specific functions of BAG3 in promoting tumor aggressiveness.

### 3.2 Comparative analysis of BAG3 protein–protein interactions in spheroid culture conditions vs adherent conditions reveals a shift from regulation of stem‐like properties to ciliogenesis

To further address the molecular processes underlying BAG3‐driven malignancy in an unbiased manner, we performed BAG3 co‐IP MS in U251 GBM cells expressing Flag‐tagged FL BAG3 in comparison to EV controls. The high pull‐down efficiency of FL BAG3‐FLAG fusion protein in U251 adherent GBM cells was verified and confirmed by Western blot (Figure S3A). In total 669 proteins were quantified by co‐IP‐MS in cells with BAG3 re‐expression, with 42 of them being significantly enriched according to the selected cut‐off criteria set (Log_2_FC >1, −log_10_ *p*‐value>1.3) (Figure S3B). The high pull‐down efficiency of FL BAG3‐FLAG fusion protein in U251 sphere GBM cells was verified and confirmed by Western blot (Figure S3C). In sphere cultured U251 cells 1045 proteins were quantified by co‐IP‐MS of FL BAG3, with 66 significant interactors (Figure S3D). Among the interactors we found BAG3 itself, as well as the known BAG3 interactor heat shock protein family B Member 8 (HSPB8), not significantly enriched were the previously reported interactors 14–3‐3y protein and the heat shock protein family B (small) member 1 (HSPB1 or HSP70).^13^

**FIGURE 2:**
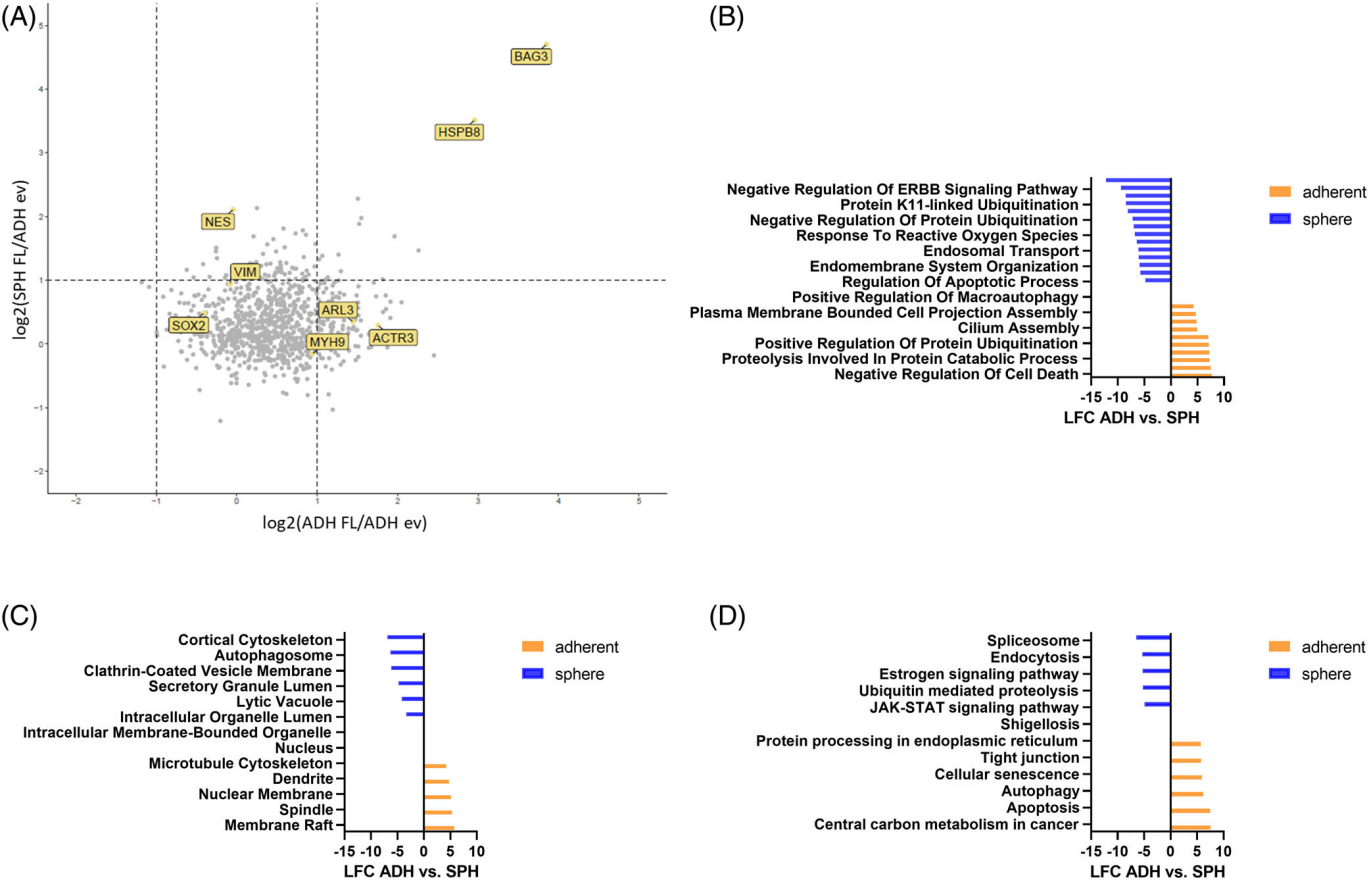
Comparative interactome analysis between adherent GBM cells and glioma stem‐like cells (GSCs) of the full‐length (FL) BAG3 U251 cell line. (A) Volcano plots of interactors that are increased in U251 FL BAG3 cells compared with EV in adherent and sphere cells. Visualized with R software. Cells were cultured for 72 h before pull‐down. (B–D) gene ontology (GO) enrichment analysis of significantly occurring interactors of the FL BAG3 U251 cell line between adherent and sphere cells. Shown in blue are the sphere‐specific and in orange the adherent‐specific GO terms. The data are shown as bar graphs. (B) Biological processes (BP). (C) Cellular components (CC). (D) KEGG pathways.

Next, we compared BAG3 protein–protein interactions in cells grown as tumor spheres and cells grown in adherent culture (Figure 2A). Interestingly, we observed a specific increase in interactors influencing primary ciliogenesis, including actin related protein 3 (ACTR3 or ARP3) and the GTPase ADP‐ribosylation factor‐like protein 3 (ARL3), and those involved in autophagy, like Myosin heavy chain 9 (MYH9), in adherent U251 FL BAG3 cells. In sphere cultured U251 FL BAG3 cells, there was a pronounced increase in BAG3 interactors that regulate the stem cell‐like character of GBM, including NES, Vimentin (VIM) and SOX2 (Figure 2A). GO‐term analysis revealed that BAG3 interacted stronger with proteins involved in cilium assembly specifically in U251 adherent cells (Figure 2B). In addition, adherent culturing conditions promoted the association of BAG3 with proteins involved in autophagy (Figure 2B–D). In cells cultured as spheres, BAG3 is associated more with proteins involved in “Negative Regulation of ERBB Signaling Pathway” and “JAK‐STAT signaling pathway” (Figure 2B–D).

### 3.3 Deletion mutants of all BAG3 domains except the WW domain decrease the number of ciliated cells

**FIGURE 3:**
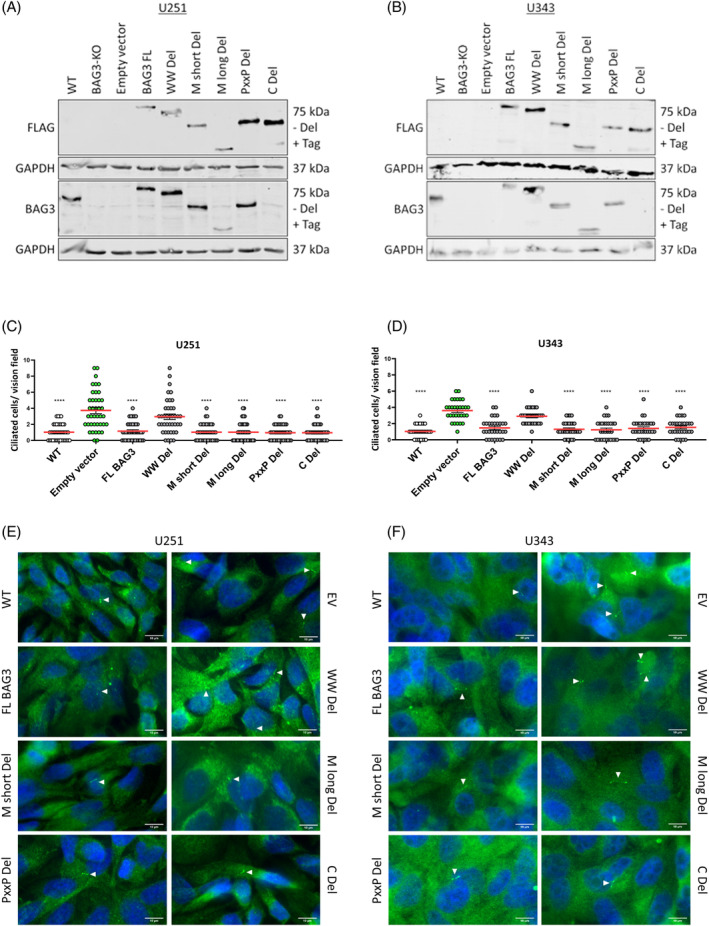
BAG3‐KO and the deletion of the WW domain leads to more ciliated cells in GBM. (A, B) Western Blot analysis of the (A) U251 +/− BAG3‐KO +/− BAG3 Del adherent and (B) U343 +/− BAG3‐KO +/− BAG3 Del adherent GBM cell lines for FLAG and BAG3 protein expression and GAPDH served as a housekeeper. (C, D) Point plots of the quantification of 10 vision fields of the IFT88 staining of four independent experiments of the (C) U251 +/− BAG3‐KO +/− BAG3 Del adherent and three independent experiments of the (D) U343 +/− BAG3‐KO +/− BAG3 Del adherent GBM cell lines. One‐way Anova with Dunnetts multiple comparisons test was performed. (E, F) Representative Images of IFT88 staining of (E) U251 +/− BAG3‐KO +/− BAG3 Del adherent and (F) U343 +/− BAG3‐KO +/− BAG3 Del adherent GBM cell lines 48 h after seeding. Cilia are shown as arrow heads. *p < 0.05; **p < 0.01; ***p < 0.001; ****p < 0.0001.

Our co‐IP‐MS data indicated an association of BAG3 with stem‐like cell‐regulatory proteins in U251 GSCs, with potential implications for the crosstalk between stem‐like properties and cilia. Previous findings by Goranci‐Buzhala et al. suggest that induction of the primary cilium leads to cell differentiation of GSCs.^60^ This finding is in line with our previous results demonstrating that depletion of BAG3 leads to an increased number of primary cilia in GBM cultures, as well as to alterations in processes related to the primary cilium in global proteomic/phosphoproteomic analysis.^23^ Of note, BAG3 is a multidomain protein containing a BAG domain, a WW domain and a PxxP domain,^14^ as well as two IPV motifs,^15^ coupling BAG3 to diverse domain‐specific interactors and biological effects. Therefore, we investigated the question which domains of BAG3 are required for suppressed formation/enhanced degradation of cilia. To this end, we applied a series of BAG3 constructs with deletion of the WW domain (WW Del), of a part of the IPV motif (M short Del), of both IPV motives (M long Del), of the proline‐rich domain (PxxP Del), and the BAG domain (C Del), in comparison to FL BAG3 in U251 and U343 cells lacking expression of endogenous BAG3 (BAG3‐KO). In addition, a pcDNA3.1 EV was introduced as a negative control. The cells were confirmed for successful transfection of the constructs and the presence of the BAG3‐KO by Western blot analysis (Figure 3A,B). Both cell lines showed a complete loss of BAG3 protein expression for the BAG3‐KO and the EV, as well as a successful re‐expression of the FL BAG3 and the individual deletion mutants. All deletion mutants and FL BAG3 have an N‐terminal FLAG‐, S‐, SBP‐, and His‐tag, resulting in a slightly larger protein size for FL BAG3 compared with WT cells for BAG3 protein expression. To gain deeper insight into which BAG3 domain is responsible for the increased cilia number, immunofluorescence staining for the individual BAG3 domain deletions was performed in cell lines U251 (Figure 3C,E) and U343 (Figure 3D,F) using the cilium marker IFT88. This approach revealed that all deletion mutants except WW Del were able to rescue the cilia suppressing effect of FL BAG3 in the BAG3‐KO background. These data suggest that the WW domain of BAG3 is required for cilia suppression in GBM.

### 3.4 The deciliating activity of YAP1 depends on the WW domain of BAG3

**FIGURE 4:**
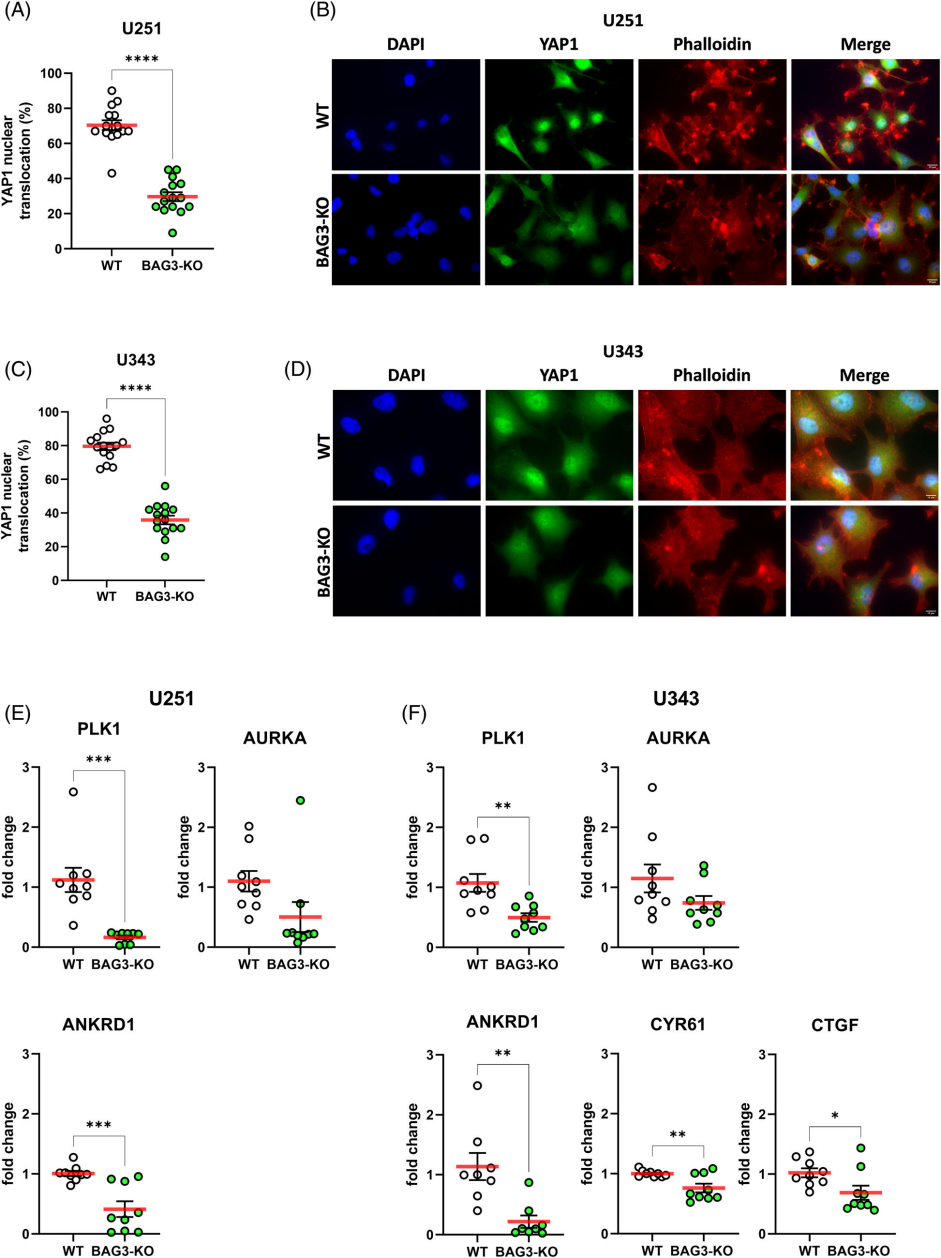
BAG3‐KO leads to less YAP1 nuclear translocation and YAP1 target gene expression. (A, C) Point plots of the quantification of YAP1 nuclear translocation in percent of five vision fields of three independent experiments. Shown are (A) U251 sph. and (C) U343 sph. Wild type (WT) and BAG3‐KO cells 48 h after seeding. Unpaired *t*‐test was performed. (B, D) Representative Images of DAPI (blue), YAP1 (green) and phalloidin (red) staining of (B) U251 sph. and (D) U343 sph. GBM cell lines 48 h after seeding. (E, F) Taqman‐based quantitative reverse‐transcription polymerase chain reaction of (E) U251 sph. and (F) U343 sph. GBM cells after BAG3‐KO of YAP1 target genes. Samples were collected after culturing in NBA medium 72 h after seeding. Unpaired *t*‐test was performed. *p < 0.05; **p < 0.01; ***p < 0.001; ****p < 0.0001.

We had shown that the loss of the BAG3 WW domain leads to a loss of function of the cilia‐suppressing effect of BAG3, but it remained to be clarified how this process is regulated. It is already known that LATS1/2 and AMOTL1/2, which are members of the Hippo signaling pathway, interact with the WW domain of BAG3.^15^, ^61^ Klimek et al. also showed that components of the Hippo signaling pathway interact with BAG3, including serine/threonine kinase 38 (STK38).^62^ In general, some cancers including brain tumors exhibit increased levels of YAP/TAZ, associated with increased activity in the cell nucleus.^25^ A connection between BAG3 and the activity of YAP/TAZ via nuclear translocation is already established.^63^, ^64^ On this basis, YAP1 nuclear translocation was analyzed in the GBM cells after BAG3 depletion in comparison to WT cells. For both GBM cell lines used, U251 (Figure 4A,B) and U343 (Figure 4C,D), a significant reduction of YAP1 nuclear translocation after BAG3 depletion was shown. The result was confirmed by investigating the respective amounts of YAP1 in nuclear versus cytoplasmic protein fractions followed by western blot (Figure S4). A significantly reduced amount of YAP1 protein content can be observed in the nuclear fractions of U251 cells in the absence of BAG3, as compared with BAG3‐proficient cells. These observations are consistent with the findings of Günay et al., who demonstrated that mechanical changes in skeletal muscle in culture are influenced by BAG3‐mediated localization of YAP and TAZ in muscle progenitor cells, while depletion of BAG3 increases the cytoplasmic retention of YAP and TAZ.^63^ To study the activity of YAP1, a qPCR for YAP1 target genes was performed. The expression of *PLK1* and *Ankyrin repeat domain‐containing protein 1* (*ANKRD1*) was significantly reduced in the cell line U251 after BAG3‐KO, while the expression of *AURKA*, another YAP1 target gene, was also suppressed (Figure 4E). The same could be shown for the cell line U343, additionally revealing a significant reduction of the two YAP1 target genes *Cysteine‐rich angiogenic inducer 61* (*CYR61*) and *Connective tissue growth factor* (*CTGF*) after BAG3 depletion (Figure 4F). In summary, our data support the hypothesis that BAG3 induces YAP1 activity and target gene expression in GBM.

**FIGURE 5:**
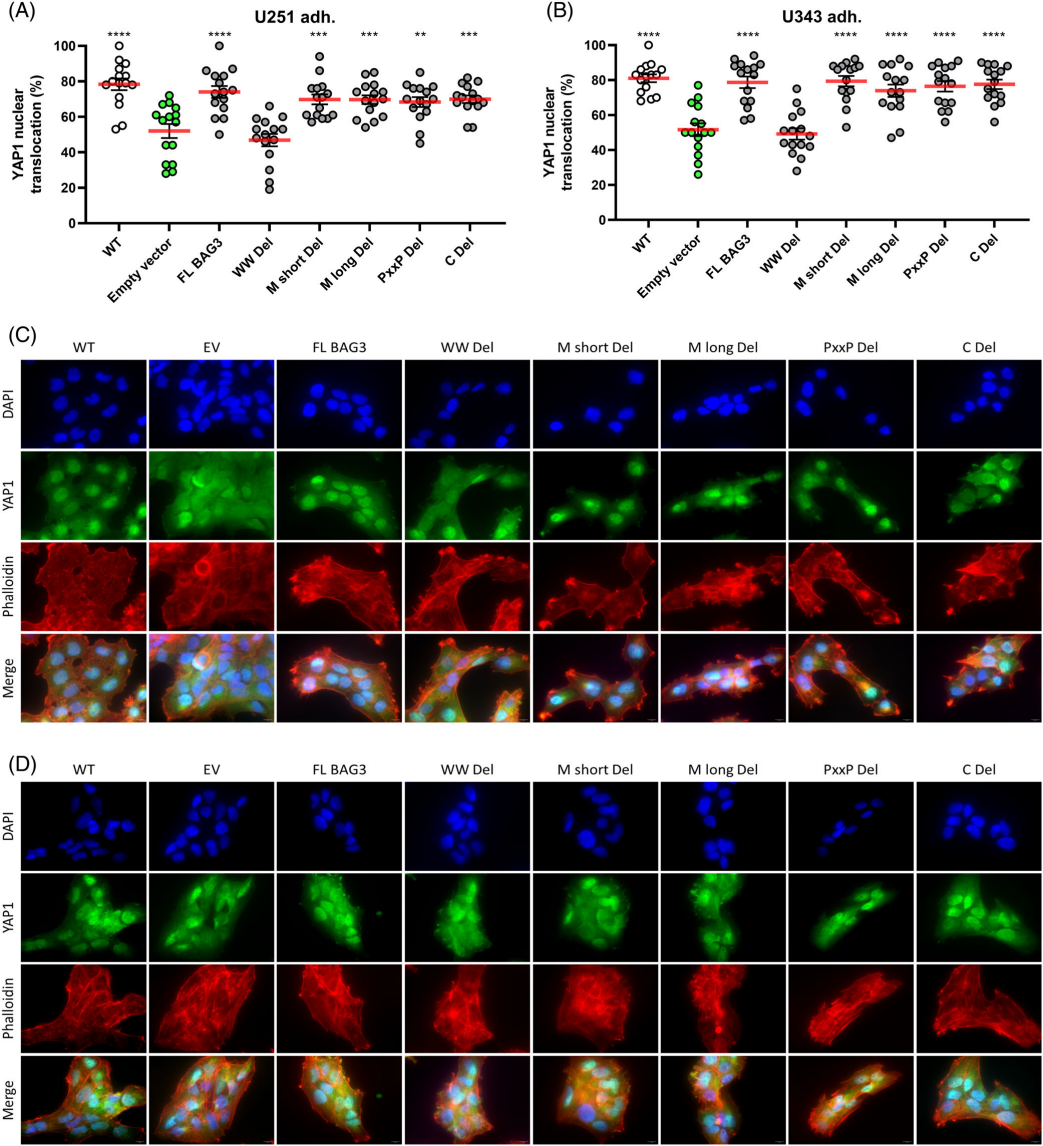
Deletion of the BAG3 WW domain leads to less nuclear translocation of YAP1. (A, B) Point plots of the quantification of YAP1 nuclear translocation in percent of five vision fields of three independent experiments. Shown are (A) U251 +/− BAG3‐KO +/− BAG3 Del adherent and (B) U343 +/− BAG3‐KO +/− BAG3 Del adherent GBM cells 48 h after seeding. One‐way Anova with Dunnetts multiple comparisons test was performed. (C, D) Representative Images of DAPI (blue), YAP1 (green), and phalloidin (red) staining. Shown are (C) U251 +/− BAG3‐KO +/− BAG3 Del adherent and (D) U343 +/− BAG3‐KO +/− BAG3 Del adherent GBM cells 48 h after seeding. *p < 0.05; **p < 0.01; ***p < 0.001; ****p < 0.0001.

Since we found that the WW domain of BAG3 mediated the suppression of ciliation, we further tested if the WW domain was required to maintain nuclear localization of YAP1. Similar to EV and the parental BAG3‐KO cell line, we could show that the loss of the WW domain in U251 GBM cells also evokes a reduced YAP1 nuclear translocation (Figure 5A,C). For the U343 GBM cell line, we could also demonstrate for the EV and WW domain deletion that significantly less YAP1 was present in the cell nucleus (Figure 5B,D), suggesting that the WW domain of the BAG3 protein seems to be responsible for the regulation of YAP1 shuttling.

### 3.5 YAP1 and HDAC6 inhibition increases the number of ciliated GBM cells

**FIGURE 6:**
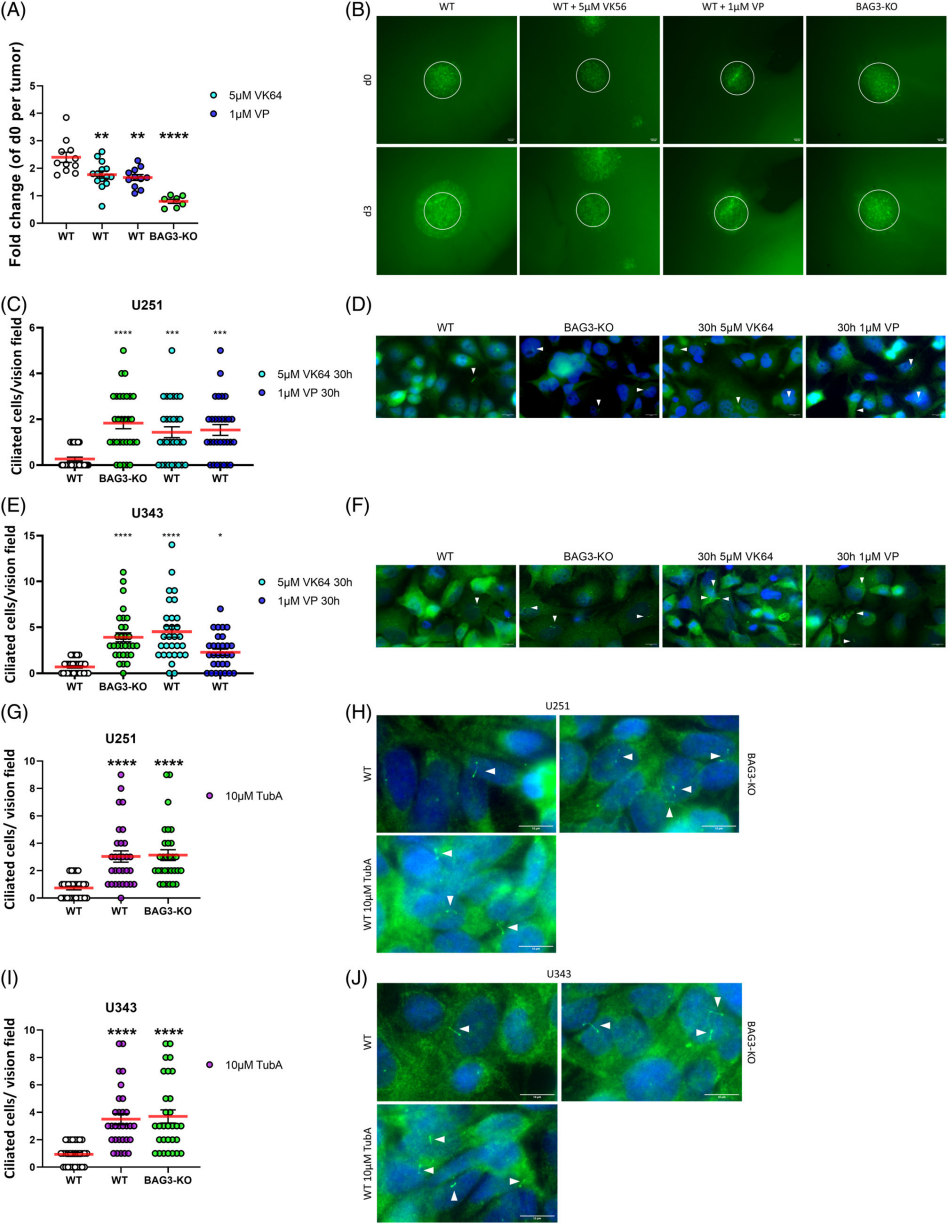
Inhibition of YAP1 leads to a reduced tumor growing capacity in the organotypic tissue culture (OTC) model and to more ciliated cells, mimicked by the inhibition of HDAC6. (A) Point plots of the quantification of at least seven tumors from OTC experiments with EGFP fluorescent U251 Wild type (WT) and BAG3‐KO cells. Additionally, WT‐transplanted cultures were treated with 5 μM VK64 or 1 μM verteporfin (VP) for 3 days. One‐way Anova with Dunnetts multiple comparisons test was performed. (B) Representative Images of EGFP fluorescent U251 WT and BAG3‐KO cells and additional treatment of WT cells with 5 μM VK64 or 1 μM VP for 3 days. Fotos were taken on days 0 and 3. The white circles represent the tumor area at day 0. (C, E) Point plots of the quantification of 10 vision fields of the IFT88 staining of three independent experiments of the (C) U251 WT and BAG3‐KO and (E) U343 WT and BAG3‐KO GBM cell lines 48 h after seeding with additional treatment with 5 μM VK64 and 1 μM VP for 30 h were indicated. One‐way Anova with Dunnetts multiple comparisons test was performed. (D, F) Representative Images of IFT88 staining of (D) U251 and (F) U343 WT and BAG3‐KO cell lines 48 h after seeding. Additional treatment with 5 μM VK64 and 1 μM VP for 30 h was done were indicated. Cilia are shown as arrowheads. (G, I) Point plot of three individual experiments with 10 vision fields per experiment of evaluation of cilia per vision field with and without 10 μM TubA (48 h) treatment of (G) U251 glioblastoma (GBM) cells and (I) U343 GBM cells 48 h after seeding. BAG3‐KO was seeded as control. One‐way Anova with Dunnetts multiple comparisons test was performed. (H, J) Representative images of IFT88 cilia staining of (H) U251 WT and BAG3‐KO cells and (J) U343 WT and BAG3‐KO cells with additional treatment of WT cells with 10 μM Tub A for 48 h. Nuclei are shown in blue. *p < 0.05; **p < 0.01; ***p < 0.001; ****p < 0.0001.

In order to transfer these findings into a more complex model, the influence of BAG3/YAP1‐signaling on tumor growth was validated in an OTC transplantation model. For this purpose, U251 GSCs were treated for 3 days with two different YAP1 inhibitors. Consistent with previous findings,^16^ genetic depletion of BAG3 led to a significant reduction in tumor sizes (Figure 6A,B), an effect that could partially be mimicked with the two YAP1 inhibitors VK64 and VP. These results support the concept that BAG3‐mediated YAP1 activation may have an important impact on the aggressiveness and growth of GBM tumors. To test whether the effects of depleted BAG3/the deleted WW domain on cilia homeostasis can be mimicked by a pharmacological approach, the YAP1 inhibitors were used again. For this purpose, both U251 WT (Figure 6C,D) and U343 WT (Figure 6E,F) cells were treated with VK64 (5 μM) and VP (1 μM) for 30 h. The treatment led to a significant increase in the number of ciliated cells in both cell lines, reaching a similar percentage as in corresponding untreated BAG3‐KO cells. These data support the hypothesis that the suppressing effect of BAG3 on primary cilia is mediated via YAP1 signaling. Moreover, they indicate that YAP1 nuclear translocation is regulated via the WW domain of BAG3, which in turn influences cilia homeostasis. The target genes *AURKA* and *PLK1* located downstream of YAP1 activity are both directly or indirectly involved in the degradation of the primary cilium. The histone deacetylase 6 (HDAC6) is activated through phosphorylation by AURKA and is known to mediate cilium degradation.^8^ Our findings obtained so far suggested reduced activity of YAP1 and suppressed YAP1 target protein expression of *AURKA* and *PLK1* after BAG3‐KO. To further corroborate the relevance of this signaling pathway in regard to the role of HDAC6, both GBM lines were treated with the HDAC6 inhibitor Tubastatin A (Tub A). In line with the established role of HDAC6 in cilia degradation, the number of primary cilia after treatment was significantly increased in the U251 WT cell line compared with the untreated WT control (Figure 6G,H). The U343 cell line showed a comparable significant increase in the number of cilia after HDAC6 inhibition (Figure 6I,J).

## 4 DISCUSSION

There is increasing evidence that the co‐chaperone BAG3 serves as a central hub molecule in cancer cell signaling, involving a diverse set of oncogenic mechanisms on the cellular level as well as in the tumor microenvironment.^13^, ^14^, ^15^ Several lines of evidence point at a key function of BAG3 in therapy‐resistant cancers such as TNBC and GBM, and we recently identified a completely novel deciliating function of BAG3 in both tumor entities,^23^ representing an interesting putative mechanism further driving the aggressiveness of these tumors. This hypothesis, that is based on the notion that cilia represent “tumor suppressor organelles” involved in controlling several hallmarks of cancer, including apoptosis resistance, EMT, cell cycle progression, oncogenic signaling, autophagy, cancer cell metabolism, and angiogenesis,^8^, ^18^, ^60^ is now further supported by the present study.

In continuation of our previous study that investigated alterations of global proteomes/phosphoproteomes in BAG3‐depleted U251 and U343 GBM cells, we now identified the BAG3 interactomes in both cell models to determine inasmuch the different oncogenic functions may be functionally coupled to BAG3‐dependent networks via direct target protein stabilization/degradation. Several important conclusions can be drawn from the obtained interactome data: consistent with BAG3's proposed role in promoting cancer stemness like properties,^33^, ^34^ we found an enrichment of the stemness regulators NES, VIM, and SOX2 in sphere‐cultured BAG3‐expressing U251 cells versus BAG3‐KO (EV) cells. In line with these observations, BAG3‐depleted U251 and U343 cells exhibited reduced sphere formation capacity, decreased stem cell frequency and decreased expression of stem cell markers as analyzed by qPCR. Collectively, these data underscore the critical role of BAG3 in maintaining stemness‐like properties of GSCs. Furthermore, many processes related to autophagy and the autophagosome appeared both in adherently and in sphere‐cultured BAG3‐proficient U251 cells, with possible functional implications for tumor stem‐like properties and cilia homeostasis (see below).

The most striking finding of the BAG3 interactome analysis was the identification of the process “cilium assembly” correlated to the enrichment of factors directly linked to cilia formation/turnover. Identified BAG3 interactors putatively involved in primary ciliogenesis include ACTR3 and ARL3. Ciliogenesis is organized by actin remodeling, where ACTR3, localized at the ciliary base, is centrally involved. If ACTR3 is present, a branched actin network is present, which inhibits ciliogenesis. Thus, depletion of ACTR3 leads to a greater length of axonemes, the axial filaments of cilia, and an increased number of ciliated cells.^65^, ^66^ Thus, ACTR3 is a negative regulator of cilium assembly. The GTPase ARL3 is known to regulate cilium assembly and trafficking of membrane associated protein complexes.^67^, ^68^ Accordingly, ARL3 is a positive regulator of cilium assembly. Mechanistically, it is currently unclear how interaction of BAG3 with these factors could affect ciliogenesis, but the cilia‐suppressing function of BAG3 would rather point to a ciliogenesis‐inhibiting function of these interactions. Autophagy and ciliogenesis are also closely linked. MYH9 is upregulated in adherent U251 FL BAG3 GBM cells and is known to inhibit ciliogenesis. The autophagic degradation of MYH9 mediated by the autophagy adapter NIMA Related Kinase 9 (NEK9) leads to increased ciliogenesis.^69^, ^70^

Importantly, cilia homeostasis is maintained by the balance of cilium assembly/disassembly, the latter process being regulated by cell cycle‐associated proteins such as AURKA and PLK1.^18^ Our previously performed phosphoproteomic analysis had revealed that the activity of AURKA and CDK1 (an upstream activator of PLK1) was strongly reduced in BAG3‐depleted cells, suggesting that BAG3 may drive enhanced cilium turnover via AURKA and CDK1/PLK1. AURKA and CDK1 are target genes of the transcription factor YAP1, a component of the Hippo signaling pathway that is coupled to the WW domain of BAG3 via LATS1/2 and AMOTL1/2.^13^, ^18^, ^24^ Consistent with the proposed role of BAG3 and its WW domain in regulating nuclear translocation of YAP1, we could demonstrate a reduced percentage of nuclear YAP1 in BAG3‐depleted GBM cells that could be rescued by re‐expression of FL BAG3 and all deletion constructs except the WW deletion construct. BAG3 has already been linked to YAP/TAZ activity by regulating nuclear translocation.^63^, ^64^ In line with these findings, we observed that all deletion mutants except the WW‐Del construct suppressed the number of ciliated cells in both GBM lines. The effect of the cilium disassembly mediator HDAC6 that is induced by phosphorylation via AURKA was shown by inhibition with Tubastatin A by mimicking of the BAG3‐KO effect by increasing cilia number. Of note, HDAC6 signaling at primary cilia was previously shown to promote proliferation and to restrict differentiation of glioma cells.^71^

Based on our data, we propose that BAG3 regulates the levels of multiple client proteins involved in cilia homeostasis, either through direct complex formation or through indirect effects mediated by respective upstream regulators. BAG3 is crucially involved in cilium disassembly/turnover, and this axis of BAG3‐signaling is at least in part mediated by activation of a transcriptional profile involving activation of the YAP1/AURKA/HDAC6 pathway and CDK1/PLK1. In parallel, BAG3 appears to be involved in regulating cilium formation by direct interaction of ciliogenesis factors on the post‐transcriptional level. Collectively, these findings suggest a complex and multilayered interaction network of BAG3 with several pathways regulating cilia homeostasis at the level of ciliogenesis and cilium degradation, providing further mechanistic insights into the diverse oncogenic roles of BAG3 in GBM and other therapy‐resistant cancers.

## AUTHOR CONTRIBUTIONS

*Conceptualization*: Donat Kögel, Benedikt Linder, and Christian Behl. *Data curation*: Caterina Roth, Marina E. Hoffmann, and Lara Paulini. *Formal analysis*: Caterina Roth and Marina E. Hoffmann. *Investigation*: Caterina Roth and Benedikt Linder. *Methodology*: Caterina Roth and Marina E. Hoffmann. *Resources*: Donat Kögel, Ivan Dikic, Andreas Brunschweiger, Hagen Körschgen, and Christian Behl. *Funding acquisition*: Donat Kögel and Andreas Brunschweiger. *Project administration*: Donat Kögel. *Software*: Caterina Roth and Marina E. Hoffmann. *Supervision*: Donat Kögel. *Validation*: Caterina Roth. *Visualization*: Caterina Roth. *Writing—original draft*: Donat Kögel and Caterina Roth. *Writing—review and editing*: Donat Kögel, Caterina Roth, Hagen Körschgen, Christian Behl, and Benedikt Linder.

## FUNDING INFORMATION

This research was funded by the German Research Foundation (Deutsche Forschungsgemeinschaft), grant numbers KO1898/11–1 to Donat Kögel and BR 5049/3–1 to Andreas Brunschweiger, and project‐ID 259130777—SFB 1177 to Donat Kögel, Christian Behl, and Ivan Dikic.

## CONFLICT OF INTEREST

There is no conflict of interest.

## Supporting information


**Supplemental Figure S1.** BAG3 depletion reduces the stemness‐like character in GSCs in vitro. (A, B) Log‐fraction plot of the limiting dilution assay of (A) U251 WT (black) and BAG3‐KO (red) cells and (B) U343 WT (black) and BAG3‐KO (red) cells. Cells were seeded in a dilution series of 1024 to 8 cells per well and analyzed after 7 days using ELDA software.^47^ Data are the summary of at least three independent experiments performed with 12 replicates per cell number. (C) Table showing the estimated stem cell frequency and statistical test of significance for (A) and (B) using chi‐square test from ELDA web app.^47^

**Supplemental Figure S2.** BAG3 depletion sensitizes GBM cells to cell death induction by the BH3 mimetic ABT‐737 and the conventional chemotherapeutic agent TMZ. OTC experiment of U251 WT and BAG3‐KO cells with additional treatment of 5 μM ABT‐737. (A) Time course of tumor size fold changes from day 0 to day 19. (B) Point plots of the quantification of at least 8 tumors per condition of the OTC experiment of EGFP fluorescent U251 WT and BAG3‐KO cells with and without ABT‐737 treatment at day 19. One‐Way Anova with Dunnetts multiple comparisons test was performed. (C) Representative Images of EGFP fluorescent U251 WT and BAG3‐KO cells and additional treatment of ABT‐737. Images were taken on day 0, day 3, day 10 and day 19. The white circles represent tumor areas at day 0. (D) Point plot of cell death analysis pooling three individual experiments with three replicates after treatment with DMSO (control) or 100 μM TMZ for 96 h of U251 WT and BAG3‐KO GBM cells. One‐Way Anova with Tukey multiple comparisons test was performed. *p < 0.05; **p < 0.01; ***p < 0.001; ****p < 0.0001.

**Supplemental Figure S3.** Interactome analysis of FL BAG3 protein versus EV in adherent and sphere U251 cells. (A, C) High pull‐down efficiency of FL BAG3‐FLAG fusion protein. Western blot analysis to verify BAG3‐FLAG pull down in U251 (A) adherent and (C) sphere cells. Transfected cells were lysed with RIPA lysis buffer and the samples were mixed with 25 μL FLAG‐Trap and were incubated for 1 h. A protein concentration of 50 μg was used for all probes. (B, D) BAG3 interaction partners that are upregulated after BAG3 re‐expression in comparison to EV. Significantly upregulated (Log_2_FC >1; −log10 p > 1.3) interactors for the U251 (B) adherent and (D) sphere cultures are shown in red/ dark gray.

**Supplemental Figure S4.** BAG3 depletion reduces nuclear translocation of YAP1 protein. Western Blot analysis of YAP1 protein expression of U251 WT and BAG3‐KO cells after subcellular fractionation (n = 1). GAPDH serves as quality control for the cytosolic fraction and LAMIN‐A/C for the nuclear fraction.
