# Supplementary figures and images for: BAG3 regulates cilia homeostasis of glioblastoma via its WW domain
Source: Biofactors. 2024 Apr 24;50(6):1113–33. doi: 10.1002/biof.2060 (PMC11627473; doi:10.1002/biof.2060)

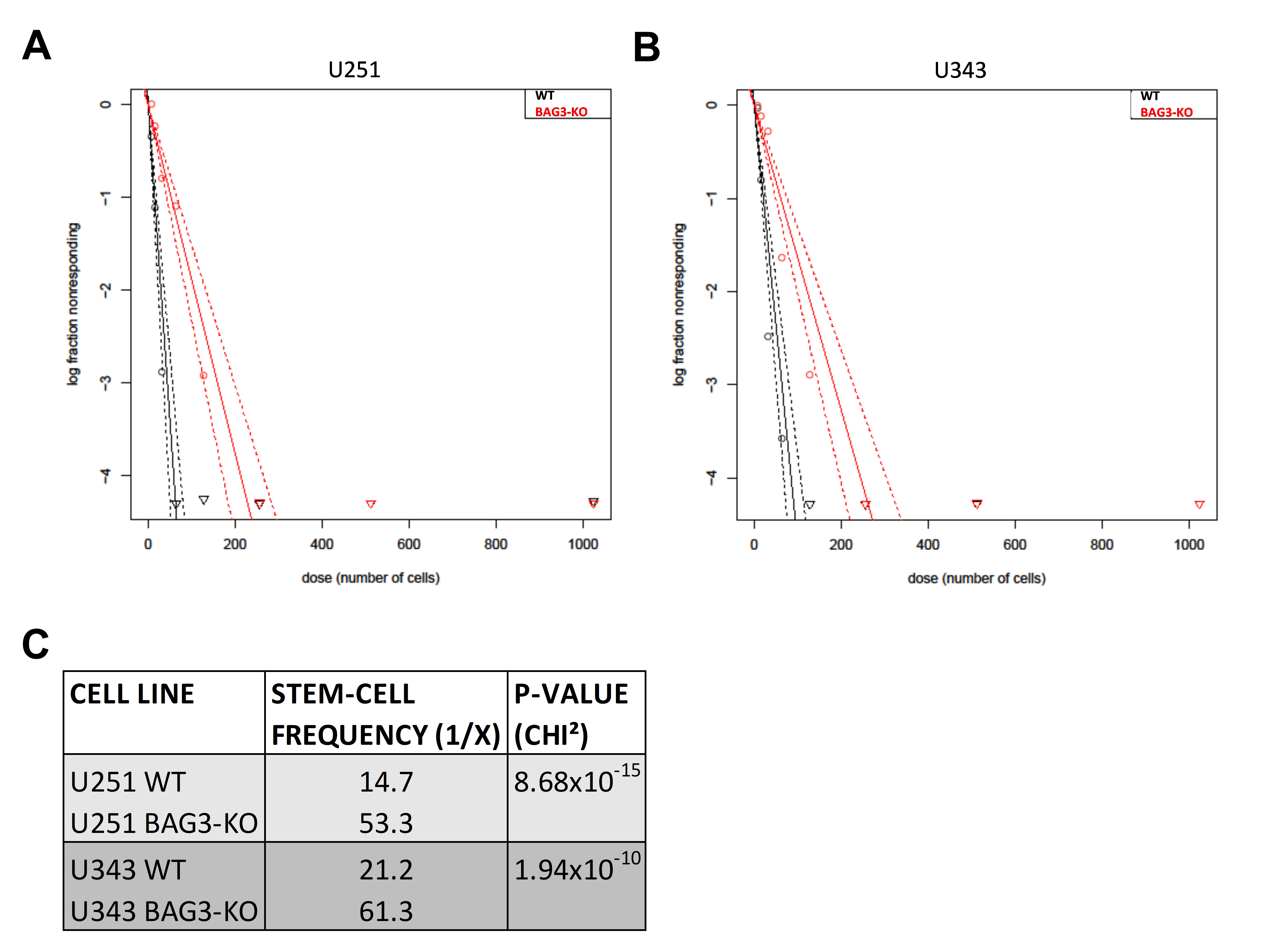

Supplement: Supplementary file 1 — Supplemental Figure S1. BAG3 depletion reduces the stemness‐like character in GSCs in vitro. (A, B) Log‐fraction plot of the limiting dilution assay of (A) U251 WT (black) and BAG3‐KO (red) cells and (B) U343 WT (black) and BAG3‐KO (red) cells. Cells were seeded in a dilution series of 1024 to 8 cells per well and analyzed after 7 days using ELDA software.47 Data are the summary of at least three independent experiments performed with 12 replicates per cell number. (C) Table showing the estimated stem cell frequency and statistical test of significance for (A) and (B) using chi‐square test from ELDA web app.47 [file BIOF-50-1113-s003.tif]

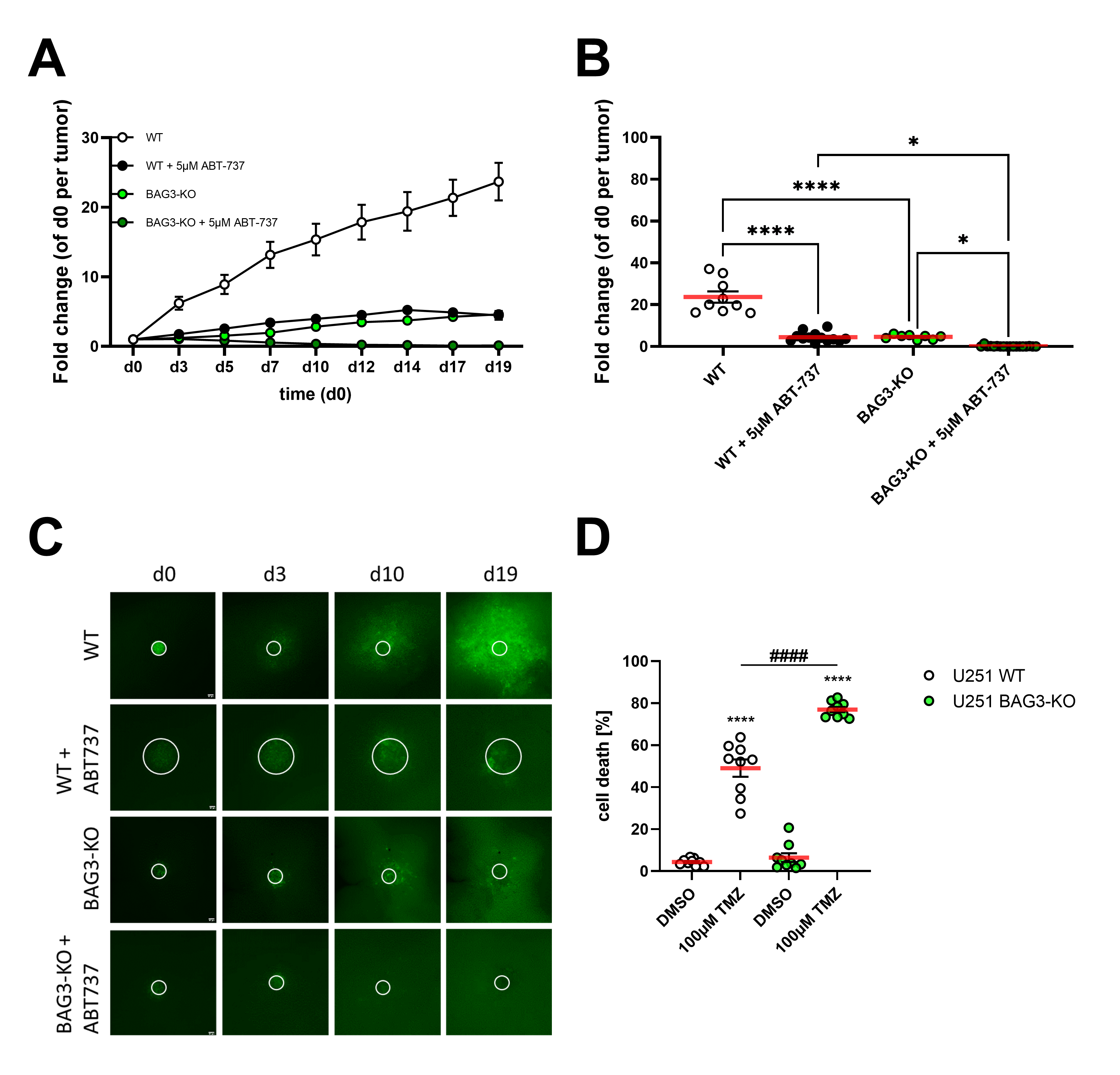

Supplement: Supplementary file 2 — Supplemental Figure S2. BAG3 depletion sensitizes GBM cells to cell death induction by the BH3 mimetic ABT‐737 and the conventional chemotherapeutic agent TMZ. OTC experiment of U251 WT and BAG3‐KO cells with additional treatment of 5 μM ABT‐737. (A) Time course of tumor size fold changes from day 0 to day 19. (B) Point plots of the quantification of at least 8 tumors per condition of the OTC experiment of EGFP fluorescent U251 WT and BAG3‐KO cells with and without ABT‐737 treatment at day 19. One‐Way Anova with Dunnetts multiple comparisons test was performed. (C) Representative Images of EGFP fluorescent U251 WT and BAG3‐KO cells and additional treatment of ABT‐737. Images were taken on day 0, day 3, day 10 and day 19. The white circles represent tumor areas at day 0. (D) Point plot of cell death analysis pooling three individual experiments with three replicates after treatment with DMSO (control) or 100 μM TMZ for 96 h of U251 WT and BAG3‐KO GBM cells. One‐Way Anova with Tukey multiple comparisons test was performed. *p < 0.05; **p < 0.01; ***p < 0.001; ****p < 0.0001. [file BIOF-50-1113-s002.tif]

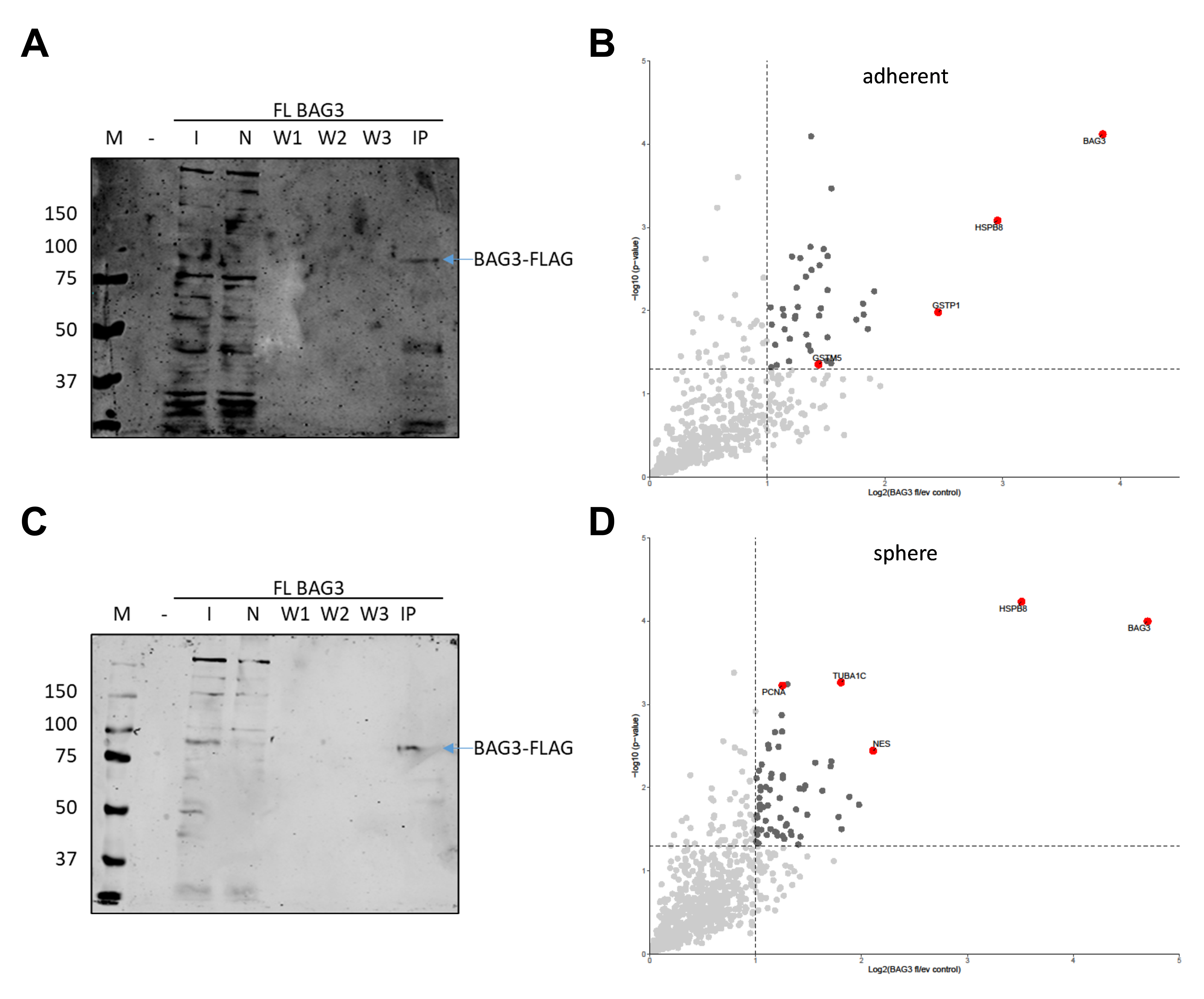

Supplement: Supplementary file 3 — Supplemental Figure S3. Interactome analysis of FL BAG3 protein versus EV in adherent and sphere U251 cells. (A, C) High pull‐down efficiency of FL BAG3‐FLAG fusion protein. Western blot analysis to verify BAG3‐FLAG pull down in U251 (A) adherent and (C) sphere cells. Transfected cells were lysed with RIPA lysis buffer and the samples were mixed with 25 μL FLAG‐Trap and were incubated for 1 h. A protein concentration of 50 μg was used for all probes. (B, D) BAG3 interaction partners that are upregulated after BAG3 re‐expression in comparison to EV. Significantly upregulated (Log2FC >1; −log10 p > 1.3) interactors for the U251 (B) adherent and (D) sphere cultures are shown in red/ dark gray. [file BIOF-50-1113-s004.tif]

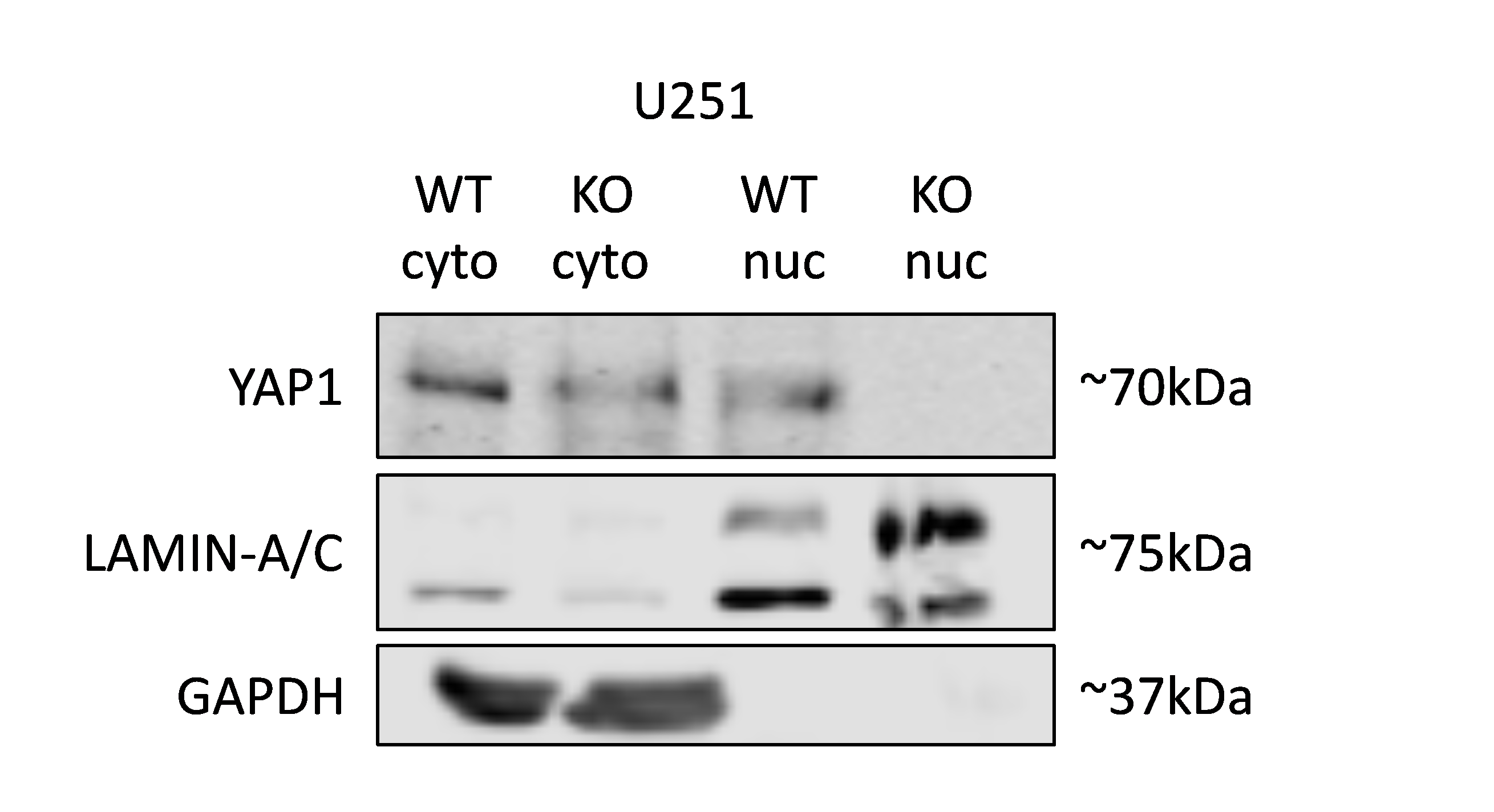

Supplement: Supplementary file 4 — Supplemental Figure S4. BAG3 depletion reduces nuclear translocation of YAP1 protein. Western Blot analysis of YAP1 protein expression of U251 WT and BAG3‐KO cells after subcellular fractionation (n = 1). GAPDH serves as quality control for the cytosolic fraction and LAMIN‐A/C for the nuclear fraction. [file BIOF-50-1113-s001.tif]
